# A lung specific escape of intravascular metastatic breast cancer cells from cytotoxic T cell killing

**DOI:** 10.3389/fimmu.2025.1599751

**Published:** 2026-01-22

**Authors:** Marina Kizner, Nehora Levi, Carmel Sochen, Julia Ryvkin, Ofer Regev, Alexander Zarbock, Lea Eisenbach, Moshe Biton, Ronen Alon

**Affiliations:** 1 Department of Immunology and Regenerative Biology, Weizmann Institute of Science, Rehovot, Israel; 2 Bioinformatics Unit, Life Science Core Facilities, Weizmann Institute of Science, Rehovot, Israel; 3 Department of Anaesthesiology, Intensive Care and Pain Medicine, University of Münster, Munster, Germany

**Keywords:** cancer immunotherapy, cytotoxic lymphocytes, adoptive cell therapy, leukocyte trafficking, breast cancer

## Abstract

The lungs are a major organ of cancer metastasis. Despite advances in the usage of tumor- specific cytotoxic T cells (CTLs) with potent killing activity (i.e., tumor infiltrating T cells, TILs) for killing of primary tumors, how these T cells encounter and kill metastatic lesions at remote organs is still poorly understood. In the present study we compared the ability of potent neoantigen specific CTLs to kill two types of cancer cells that share the same neoantigen and generate distinct metastatic lesions in the lungs of immunocompetent recipient mice. We have used ovalbumin (OVA) as a neoantigen model and found that the OVA-specific OT-I transgenic CD8 CTLs, when intravenously introduced, readily eliminated primary tumors of OVA-expressing breast cancer E0771 cells. Nevertheless, similar OT-I CTLs failed to clear OVA-expressing breast cancer E0771 cells that colonized the lungs. In contrast, similar intravenously introduced OT-I CTLs efficiently eliminated lung metastatic OVA-expressing B16 melanoma cells, ruling out that the intravenous CTLs were exhausted upon entering the lungs. Three-dimensional (3D) imaging of whole lungs revealed that in both experimental and spontaneous metastasis models, the OVA E0771 cells survived inside lung blood vessels but did not recruit circulating OT-I CTLs to their vicinity. Furthermore, canonical vascular adhesion molecules recognized by the CTLs like ICAM-1 and VCAM-1 were not upregulated nearby the lung-residing intravascular E0771 cells as a potential means to recruit lung circulating CTLs to the vicinity of the intravascular tumor cells. Strikingly, the lung residing OVA-expressing E0771 cells lost expression of their OT-I specific OVA-derived SIINFEKL-H-2Kb pMHC complexes while retaining MHC-I expression. This loss was accompanied by a lung-specific transcriptional reduction of key regulators of MHC-I presentation. A temporal loading of OVA-derived SIINFEKL-H-2Kb pMHC complexes on E0771 cells did not result, however, in cancer cell killing inside the lungs. Nevertheless, direct and stable SIINFEKL peptide presentation on these cells overcame their lung specific escape from OT-I mediated killing. Our study is a first indication that subsets of cancer cells that reside in the lungs rapidly downregulate the expression of neoantigen derived peptide MHC-I complexes and thereby evade killing by intravenously introduced tumor antigen-specific CTLs.

## Introduction

Breast cancer is the most common malignant disease in women worldwide (1). Despite remarkable improvements in cancer survival rates, distant metastasis continues to account for nearly 90% of cancer-related deaths (2). Considerable progress in breast cancer therapies has been achieved with antibody-based immunotherapies such as immune checkpoint blocking therapies targeting both primary tumors and metastatic lesions (3–8). Cellular-based immunotherapies based on adoptive cell transfer (ACT) of *ex vivo* manipulated patient effector leukocytes has also provided great promise for cancer treatments. Patient T cells tailored with chimeric antigen receptor specific for a common tumor antigen (CAR-T cells) or *ex vivo* expanded tumor-infiltrating tumor-antigen specific lymphocytes (TILs) (3–5, 9, 10) have emerged as tumor-specific killer lymphocytes (6, 7). TIL therapy utilizes not only the tumor-killing effector functions of the tailored CTLs, but also their migratory properties that resemble those acquired by tumor specific effector T cells differentiated and educated inside tumor draining lymph nodes (11–13).

Metastatic lesions of primary solid tumors are composed of highly invasive derivatives of the tumors, which can share neoantigens with the primary tumors they are derived from (14, 15). Patient TILs isolated from primary tumors can therefore be useful to target and kill metastatic lesions derived of these tumors as means of personalized cancer therapy (16, 17). However, the clinical use of TILs in immunotherapy of metastasis rather than of primary tumors is still in its infancy (18). Even in cases where tumor-specific T cells successfully home to solid tumors, the capacity of these T cells to remove metastatic lesions in remote organs is still questionable (19). Thus, it is still unclear how efficiently TILs with a given neoantigen specificity can home into distant organs populated with distinct metastasis and eliminate cognate neoantigen expressing metastatic cells. This therapeutic challenge is also compounded by dynamic immunosuppressive metastasis microenvironments that actively impede the potential of ACT for anti-metastasis therapy (20).

The lungs are one of the primary sites for metastasis, and a vast majority of cancers, including breast, skin (melanoma), colon, gastric, pancreatic, and kidney cancers, can spread and colonize this highly vascularized organ (21, 22). Macro-metastatic lesions are poorly vascularized and thus present significant treatment challenges for intravenously (i.v.) injected CTLs. In contrast, micro-metastatic lesions are surrounded by a dense network of blood vessels comprising the lung vasculature and are therefore more promising targets for ACT. Recent findings in our laboratory also suggest that certain cancer cells reaching the lungs reside and proliferate within lung vasculature, giving rise to hematogenous metastasis (23). We therefore decided to test if systemic circulating TIL like CTLs with a given neo-antigen specificity can target and eliminate distinct micro-metastatic lesions in the lung in an antigen-specific manner. To that end we have set up an experimental immunocompetent murine lung metastasis model in which the ability of OVA specific TIL like CD8 T cells to encounter and kill OVA-expressing breast cancer or melanoma tumor cells could be determined.

The ability to image specific interactions of cytotoxic T cells adoptively transferred into recipient mice by real time videomicroscopy of lung adapted chambers is very limited (24, 25). This is due to the very small volume of the lung tissues that can be imaged by these methods. Conventional immunohistochemistry of thin sections is an option but in the case of rare metastatic lesions it requires a collection of numerous lung sections (26). We therefore took a completely different imaging approach, 3D imaging of whole lung lobes performed by light sheet fluorescence microscopy (LSM) of lipid-cleared lungs. This approach allows to scan whole lung lobes at high spatial resolution and thereby identify rare encounters between intravenously injected fluorescently labeled CTLs and fluorescent labeled metastatic cell residing in different lung compartments (27).

Our results demonstrate that intravenously injected OVA specific OT-I CTLs readily kill OVA-B16 residing in the lungs. Yet, identically introduced tumor antigen specific CTLs could not encounter and kill OVA-expressing E0771 breast cancer cells that accumulated inside the lungs. Intriguingly, a major fraction of these breast cancer cells rapidly lost surface expression of their OT-I binding SIINFEKL-H-2Kb pMHC complex. Surprisingly, this loss was lung specific, since at the primary site of breast tumors, OVA-expressing E0771 cells retained high OT-I binding pMHC expression and were effectively eliminated by cognate OT-I CTLs. Transcriptomic analysis of E0771 cells isolated from primary tumors and metastatic cells accumulating in lungs indicated downregulated transcription of multiple antigen processing and MHC-I presentation components in lung residing E0771 cells. Interestingly, while a temporal loading of SIINFEKL peptide on the OVA-expressing E0771 cells did not promote their susceptibility to killing by intravenously injected OT-I CTLs, a stable expression of this peptide rendered E0771 highly sensitive to OT-I mediated killing in the lungs. Our results are a first demonstration that metastatic breast cancer cells accumulating inside the lung vasculature can evade CTL mediated killing by a rapid lung specific loss of neoantigen processing and pMHC presentation.

## Materials and methods

### Murine cell lines

Murine breast adenocarcinoma cells (E0771) were purchased from CH3 Biosystems (SKU: 94A001) and grown in DMEM supplemented with 10% FBS, 1 mM sodium pyruvate, 10 mM HEPES, and 1% Penicillin-Streptomycin-Amphotericin B solution. Tomato labeled OVA-expressing B16F10 (herein OVA B16 cells) were a gift from Guy Shakhar (28) and were grown in DMEM supplemented with 10% FBS and 1% Penicillin-Streptomycin-Amphotericin B solution.

### Preparation of OT-I effector T cells

Murine CD8 effector T cells were generated from T cells isolated from the spleens of OT-I mice. Spleen-derived splenocytes were stimulated *in vitro* with SIINFEKL for 72 hours and expanded in Interleukin-2 (IL-2)-rich media for additional 4 days as described (28).

### Generation of OVA expressing E0771 cells

Red fluorescence protein (RFP) expressing E0771 cells were generated as described (29) and were periodically sorted according to their RFP expression. The RFP E0771 cells were further infected with a pHR OVA/p2a/mCherry-CaaX lentiviral vector (Addgene, cat. 113030). Cells were sorted by their surface expression of the OVA SIINFEKL peptide complexed with cell surface MHC-I (H-2Kb) detected by 25-D1.16 mAb staining (30).

### Generation of OT-I-II E0771 cells

To stably express the two OVA immunodominant peptides recognized, respectively by the OT-I and OT-II TCRs in E0771 cells, we designed a new cloning vector in which the full OVA gene (i.e., pHR OVA/p2a/mCherry-CaaX whose expression is driven by the SFFV promoter (Addgene, cat. 113030)) was replaced with tandem repeats of the two OVA immunodominant peptides, SIINFEKL and ISQAVHAAHAEINEAGR followed by an mCherry reporter protein. To incorporate the two peptide encoding sequences into the lentiviral vector, a 216 bp OT-I/II encoding fragment was assembled by PCR using six overlapping oligonucleotides (three forward and three reverse), covering the full sequence (**Supplementary Figure 1**). The lentiviral vector was stably infected into wild-type E0771 cells to generate a new breast cancer cell line, designated E0771- OT-I/II.

### Analysis of surface molecules

For analysis of cell surface molecule expression, E0771 cells were stained in FACS buffer (PBS supplemented with 2% bovine serum albumin (BSA) and 5 mM EDTA with monoclonal antibodies specific to MHC-I (H2K-b) (Clone- AF6-88.5), MHC-I (H2Db) (Clone- KH95) and to SIINFEKL bound to H2K-b (Clone 25-D1.1632), as well as PD-L1 (Clone-10F.9G2), all from Biolegend. OT-I T-cells were gated using CD45^+^ (Clone-30-F11), CD3^+^ (Clone-17A2), CD8^+^ (Clone-53-6.7), CD4^-^ (Clone-GK1.5), CD44^+^ (Clone- IM7), CD62L^-^ (Clone-MEL-14), all purchased from Biolegend, and analyzed using monoclonal antibodies specific to: CXCR3 (Clone- FAB1685P), CCR4 (Clone-2G12), CXCR4 (clone- L276F12), α4 (CD49, Clone-9C10), β1 (CD29 Clone- HMb1-1), β7 (CD80 Clone- FIB504), αL (CD11a, clone- M17/4), β2 (CD18, Clone- M18/2), CD103 (Clone-2E7) and CXCR6 (Clone-SA051D1), all from Biolegend. Background stainings were determined with matched fluorescence labeled isotype control mAbs. Cell surface staining was analyzed in a CytoFLEX flow cytometer (Beckman Coulter) and analyzed using FlowJo software v.10.7.1 (Tree Star).

### *In vitro* killing of OVA expressing E0771 cells

10^4^ OVA expressing E0771 cells were seeded in a 96 well plate and cultured overnight (O.N.). 5*10^3^-3*10^4^ OT-I effector CTLs were labeled with carboxyfluorescein succinimidyl ester (CFSE) and overlaid on the tumor cells at different effector to target ratios. One day later, the number of viable E0771 cancer cells was determined by FACS as described (29).

### Orthotopic tumor experiments and *in vivo* killing by OT-I CTLs

Mice were maintained in a pathogen-free facility, and all animal procedures were approved by the Animal Care and Use Committee of the Weizmann Institute of Science. 8- to 12-week-old female mice were used in all orthotopic tumor models. For *in vivo* killing analysis 1x10^3^ OVA-RFP E0771 cells (suspended in 50 μl of Matrigel^®^ Corning Life Sciences, cat. WBD-356234, diluted 2-fold in PBS) were implanted in the mammary fat pad. 3 days later, effector OT-I CTLs were injected i.v. (retro-orbitally) and tumor size was measured throughout the duration of the experiment, by caliper measurements (31) of length (L), width (W). and the tumor volume (V) was calculated using the formula: $V=\frac{\text{L }\times \text{ W }\times \text{ W}}{2}$.

### Light-sheet microscopy of lungs

Mice were injected with 5-(and-6)-(((4-chloromethyl)benzoyl)amino)tetramethylrhodamine) (CMTMR)- or RFP- OVA expressing cells as described in the sections below. Immediately after sacrifice, mice were transcardially perfused with PBS, and the lungs were inflated via the trachea with low gelling agarose (Cat. A9045, Sigma-Aldrich), fixed with paraformaldehyde (4% in PBS) for 2h, dehydrated and cleared using ethyl cinematic as described. Cleared lung lobes were imaged in an Ultramicroscope II (LaVision BioTec) operated by the ImspectorPro software (LaVision BioTec) as described (32). CD31 positive vessels were labeled 15 min before mice were sacrificed by intravenous injection of 6 µg of Alexa-647 conjugated anti-CD31 mAb (Biolegend, cat. 102516, clone MEC13.3). Three-dimensional rendering of LSM was performed with Imaris software (Oxford Instruments, Abingdon, Oxfordshire, UK) as described in previous sections. Surfaces of RFP or CMTMR-labeled tumor cells were created using volume and intensity as defining features to unequivocally separate them from background signals. Each cell was individually segmented, and its distance was measured with respect to the CD31-labeled blood vessels. Cell positions of intravascular were determined by this spatial analysis. The distance between tumor cells and vessels was determined using the shortest distance calculation.

### Experimental lung metastasis of OVA E0771 cells

Experimental metastases were generated by retro-orbitally injecting 1x10^6^ OVA-RFP E0771 cells. Mice were sacrificed 72 hrs later by administration of sodium pentobarbital (200 mg/Kg), and lungs were harvested and prepared for FACS analysis after transcardial perfusion with PBS. Lungs were extracted, minced and incubated in RPMI-1640 containing collagenase type 4 (Roche, cat. 10104159001, 1.5 mg/ml) and DNase I (20 µg/ml) at 37°C for 40 min. The total lung cell suspensions were transferred through a 100 µm cell strainer and centrifuged at 200 x g for 5 min at 4°C. RBCs were subsequently lysed and cells were re-suspended in ice-cold FACS buffer, filtered through a 70 µm strainer and analyzed using a CytoFLEX flow cytometer. The RFP label of E0771 cells was readily distinguishable from background fluorescence of lung cells.

### Spontaneous lung metastasis of OVA E0771 cells analyzed by LSM

For spontaneous metastasis, 3x10^5^ OVA RFP tumor cells (suspended in Matrigel^®^, 1:1 in 50 μl PBS solution) were implanted in the mammary fat pad of recipient female mice. 12 days later, tumors were surgically removed from anaesthetized mice. Mice were left intact or injected with OT-I CTLs. 3 days later Alexa-647 labeled anti CD31 was injected intravenously and 5 mins later mice were sacrificed, and lungs were harvested and processed for LSM analysis as described in previous sections. Three-dimensional rendering of LSM was performed with Imaris software (Oxford Instruments, Abingdon, Oxfordshire, UK) as described in previous sections.

### LSM analysis of CTL recruitment by metastatic E0771 cells

For analysis of CTL encounter of metastatic E0771 cells in the lungs, experimental lung metastasis of CMTMR labeled OVA-RFP E0771 was induced by tumor cell injection in the retro-orbital sinus of recipient mice as described in previous section. 3 days later 10^7^ CFSE labeled OT-I CTLs were injected intravenously and at variable time windows mice were sacrificed, transcardially perfused with PBS, and the lungs were inflated via the trachea with low gelling agarose (Sigma-Aldrich, cat. A9045), fixed with paraformaldehyde (4% in PBS) for 2h, dehydrated and cleared using ethyl cinematic as described. Cleared lung lobes were imaged in an Ultramicroscope II (LaVision BioTec).

### Experimental lung metastasis of B16 cells

0.2x10^6^ OVA B16 cells were injected into the retro-orbital veins of recipient mice and mice were sacrificed 14 days later, transcardially perfused with PBS and the lungs were extracted and stored in 4% PFA for 24 hours followed by incubation in 1% PFA for additional 24 hours. Paraffin embedding and H&E staining of 5 µm-thin sections were performed by the histology core unit of the Weizmann Institute of Science. Sections were digitalized using a Panoramic SCAN II (3DHISTECH) and analyzed using CaseViewer software (3DHISTECH). B16 metastasis burden was also determined by lung harvesting and FACS analysis of total lung cell suspensions as described for E0771 cells.

### Cell sorting for qPCR and SMARTSEQ2

10^6^ OVA-RFP E0771 cells were introduced i.v., and 3 days later fluorescent cells were sorted from whole lungs using FACS Aria III Cell Sorter. Cells were collected in the presence of Actinomycin D (Sigma-Aldrich, cat. A1410) in order to minimize *ex vivo* RNA transcription. RNA was purified from the sorted cells using the QIAGEN Rneasy kit. A second experimental group consisted of 10^3^ OVA- RFP E0771 cells implanted in mammary fat pads to generate primary tumors. Cells from primary tumors were similarly isolated and sorted on day 14 post implantation.

### qPCR RNA preparation

cDNA libraries were prepared with the High-Capacity cDNA Reverse Transcription Kit (Applied Bioscience). qPCR was performed using the QuantStudio 6 Flex Real-Time PCR System (Applied Bioscience).

### SMARTSeq2 RNA preparation

Libraries were prepared using a modified SMART-Seq2 (33). RNA lysate clean-up was performed using RNAClean XP beads (Agencourt), followed by reverse transcription with Maxima Reverse Transcriptase (Life Technologies) and whole-transcription amplification (WTA) with KAPA HotStart HIFI 2 × ReadyMix (Kapa Biosystems) for 18 cycles. WTA products were purified with Ampure XP beads (Beckman Coulter), quantified with Qubit dsDNA HS Assay Kit (ThermoFisher) and assessed with a high-sensitivity DNA chip (Agilent). RNA-seq libraries were constructed from purified WTA products using Nextera XT DNA Library Preperation Kit (Illumina). The libraries were sequenced on an Illumina NextSeq 500/550.

### SMARTSeq2 Bioinformatics pipeline methods

Paired-end reads with a median depth of 10,662,484 reads per sample were analyzed using the User-Friendly Transcriptomic Analysis Pipeline (UTAP) (34). Reads were trimmed using Cutadapt (35) v4.1. Custom mouse genome (mm10) with Gencode annotations was supplemented with chicken Ovalbumin and mCherry genes. Reads were mapped using STAR (36) v2.7.10a. The pipeline quantifies the RefSeq annotated genes. The annotation version and date are evidence-based annotation of the mouse genome (GRCm38), version M25 (Ensembl 100). Further analysis was conducted for genes having a minimum of 5 reads in at least one sample. Normalization of the counts and differential expression analysis was performed using DESeq2 (37) v1.36.0. Raw P values were adjusted for multiple testing using Benjamini and Hochbergstatistics (38). Interactive plots for each pairwise comparison were performed using Glimma v2.6.0.

Differential gene expression was calculated by a comparison of the data of the lung metastasis cells with the primary tumor cells. The criteria for significance were as follows:

- padj ≤ 0.05.
- |log2FoldChange| ≥ 1.
- baseMean ≥ 5.

The pipeline was constructed using Snakemake (39) v7.14.0.

A heatmap of the scaled z-score and rlog-transformed normalized read counts was created using pheatmap version 1.0.12 R Version 4.4.2. The top genes were selected based on the adjusted P-values.

Pathway Enrichment Analysis was performed using Metascape (http://metascape.org) (40) with the list of differentially expressed genes as input. We used default settings with a minimum overlap of 3, a minimum enrichment of 1.5, and a p-value cutoff of 0.05.

### Statistical analysis

Data in graphs are represented as mean or mean ± standard error of the mean (SEM). Students’ two-tailed unpaired t test or Mann-Whitney two-tailed U test were used to determine the significance of the difference between means of two groups. One or two-way ANOVA tests were used to compare means among three or more independent groups/categories. Significance was set to p< 0.05. Statistical details of experiments can be found in the figure legends.

## Results

### OVA RFP co-expressing E0771 cells are eliminated by cognate OT-I CTLs inside primary tumors but escape killing by the same CTLs once residing in the lungs

We have recently described several models of primary breast cancer and metastasis to lungs of the C57BL/6 breast cancer cell line E0771 (29, 41, 42). In order to follow the fate of these cells within various organs of recipient mice subjected to adoptive cell transfer (ACT) with OVA specific OT-I CTLs (43), we introduced OVA and RFP encoding vectors into these cells and isolated E0771 cells expressing high and uniform expression of surface MHC-I molecules as well as of the immunodominant OVA peptide SIINFEKL complexed with the H2Kb MHC-I variant, a pMHC recognized with high affinity by the OT-I TCR transgene (43) and detected with a specific mAb, 25-D1.16 (30) (**Supplementary Figure 2**).

**Figure 1:**
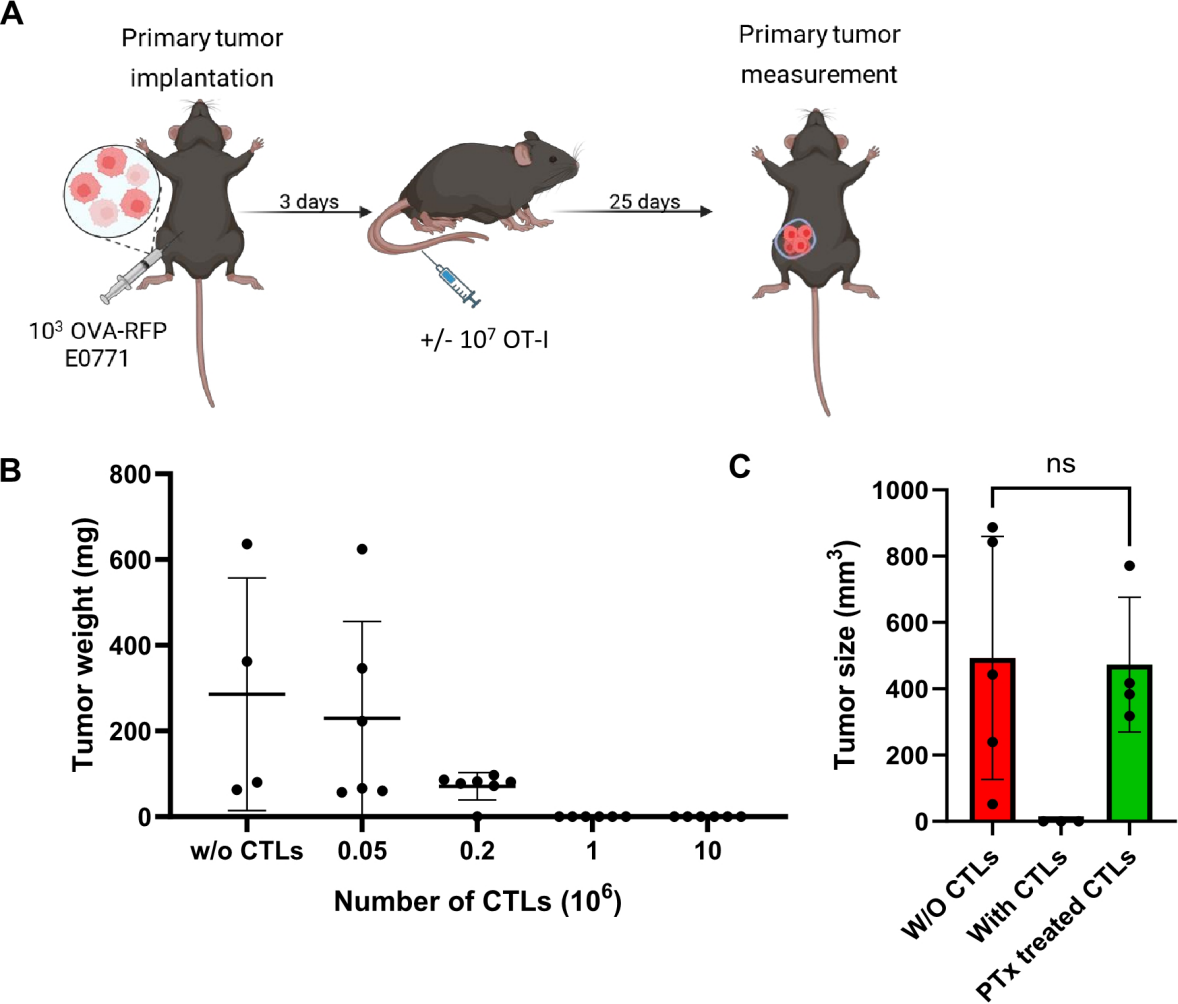
OVA-RFP E0771 cells form primary tumors in the mammary fat pads and are specifically killed by OT-I CTLs via Gi-protein signaling. **(A)** A scheme depicting the experimental procedure. WT mice were implanted with small primary tumors (1,000 cells) into the mammary fat-pad, then 3 days later different amounts of OT-I T cells were intravenously (i.v.) injected into the mice. **(B)** Dose response killing of primary OVA-RFP E0771 tumors by increasing numbers of injected OT-I CTLs. Tumors were implanted as described in A, followed by i.v. injection of the indicated numbers of OT-I CTLs. Mice were sacrificed 25 days after CTL injection and tumor size was determined by weight. Scatter plots are shown. n= 4–6 mice per each CTL treatment. W/O, without. **(C)** Tumor size determined after injection of intact or pertussis toxin (PTx)-pretreated 1x10^7^ OT-I CTLs on day 3 post tumor implantation. Mice were sacrificed 18 days after CTL injection. n=5 for each experimental category.

We next confirmed that our OVA RFP expressing E0771 cells, but not their parental E0771 counterparts, are readily targeted by *ex vivo* generated IL-2 expanded OT-I CTL effectors derived from the OT-I CD8 transgene (**Supplementary Figure 2**). To confirm the *in vivo* immunogenicity of our OVA-RFP E0771 cells, we orthotopically implanted them in the mammary fat pad of syngeneic mice and 3 days later injected *in vitro* generated OT-I CTLs into these mice and monitored tumor size for 25 days (**Figure 1A**). As expected, OVA-RFP E0771 tumors were readily eliminated by the injected OT-I CTLs in a dose dependent manner (**Figure 1B**). As reported for other systems, these effector CTLs expressed a variety of inflammatory chemokine receptors (GPCRs) and elevated levels of both β1 and β2 integrins (**Supplementary Figures 3**, **4**) involved in T cell interactions with blood vessels and extravascular ECM components at sites of inflammation (44–46). To confirm the involvement of chemotactic signals in OT-I mediated killing of our primary OVA-RFP E0771 tumors, we next pretreated OT-I CTLs with pertussis toxin (PTx), a potent inhibitor of G-protein coupled receptor signaling critical for chemotaxis and T cell trafficking across inflamed blood vessels (47). Notably, PTx-treated OT-I CTLs failed to eliminate the primary OVA-RFP E0771 tumors even at saturating doses of transferred CTLs (**Figure 1C**). Since the GPCR blocking effects of the PTx pretreatment of OT-I CTLs are non-toxic, these observations suggest that OT-I CTLs require intact GPCR machinery to accumulate at the primary site of implanted OVA RFP expressing E0771 tumors and kill their tumor targets.

**Figure 2:**
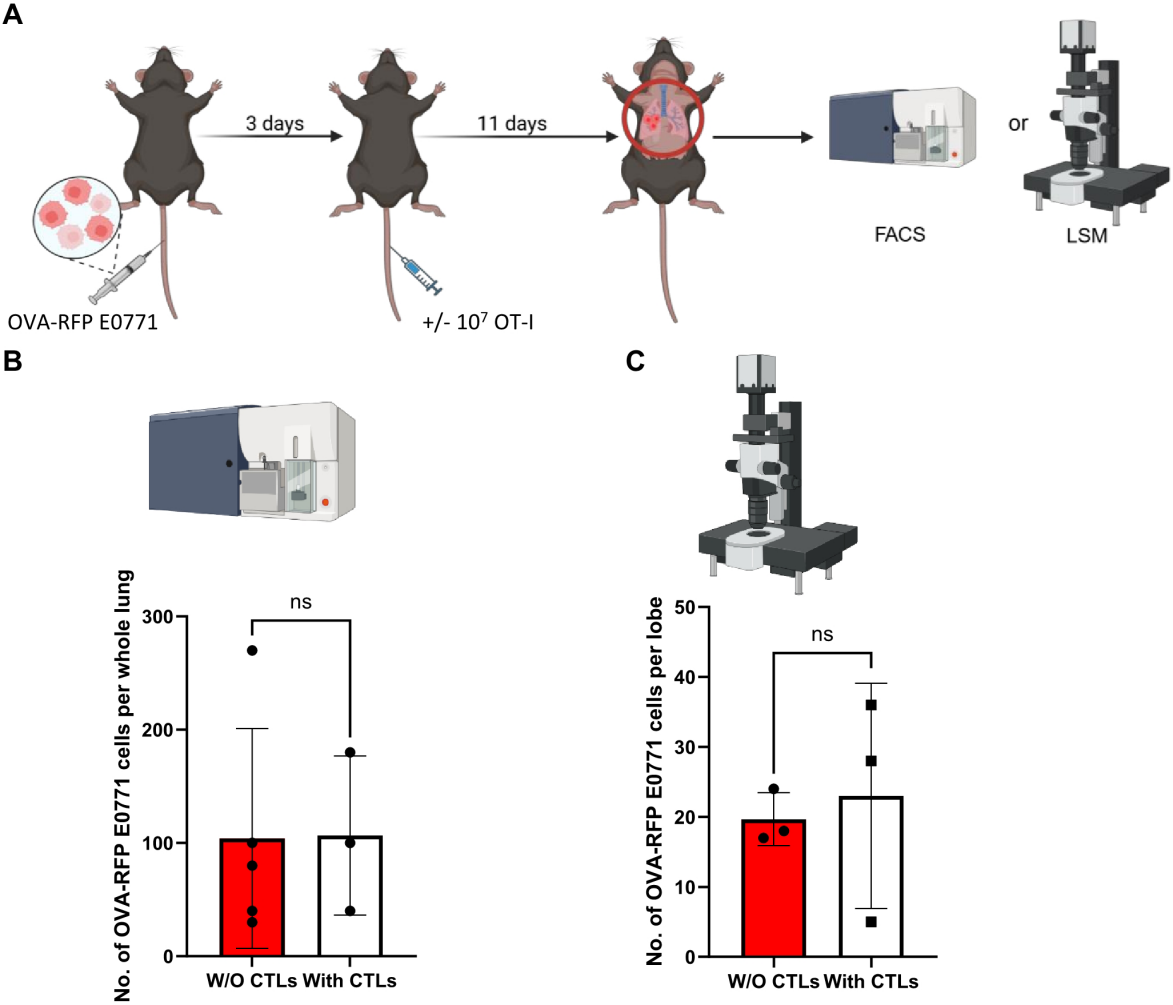
OVA specific CTLs fail to kill OVA expressing RFP E0771 in an experimental lung metastasis model. **(A)** A scheme of the experimental lung metastasis model and CTL treatments. 5x 10^4^ OVA-RFP E0771 cells were injected into C57BL/6 recipient mice and 3 days later 10^7^ OT-I CTLs were injected i.v. Control mice injected with the same E0771 cells were left without OT-I injection. 11 days later mice were euthanized, lungs were harvested, and total lung cell suspensions were prepared for FACS analysis. In a separate line of experiments, mice were injected i.v. with Alexa-647 labeled anti-CD31 i.v. 5 mins before euthanasia and harvested lungs were fixed and processed for LSM analysis. **(B)** The number of OVA-RFP E0771 cells recovered in whole lungs, analyzed by FACS after OT-I CTL treatment. n=3. The scatter plots and bars depict the mean values ± SEM. ns= non-significant. **(C)** The effect of i.v. injected OT-I CTLs on the number of OVA expressing RFP E0771 remaining in the lungs as imaged by LSM. 3 lobes from 3 mice were analyzed in this experiment. The bars depict the mean values ± SEM. ns, non-significant.

**Figure 3:**
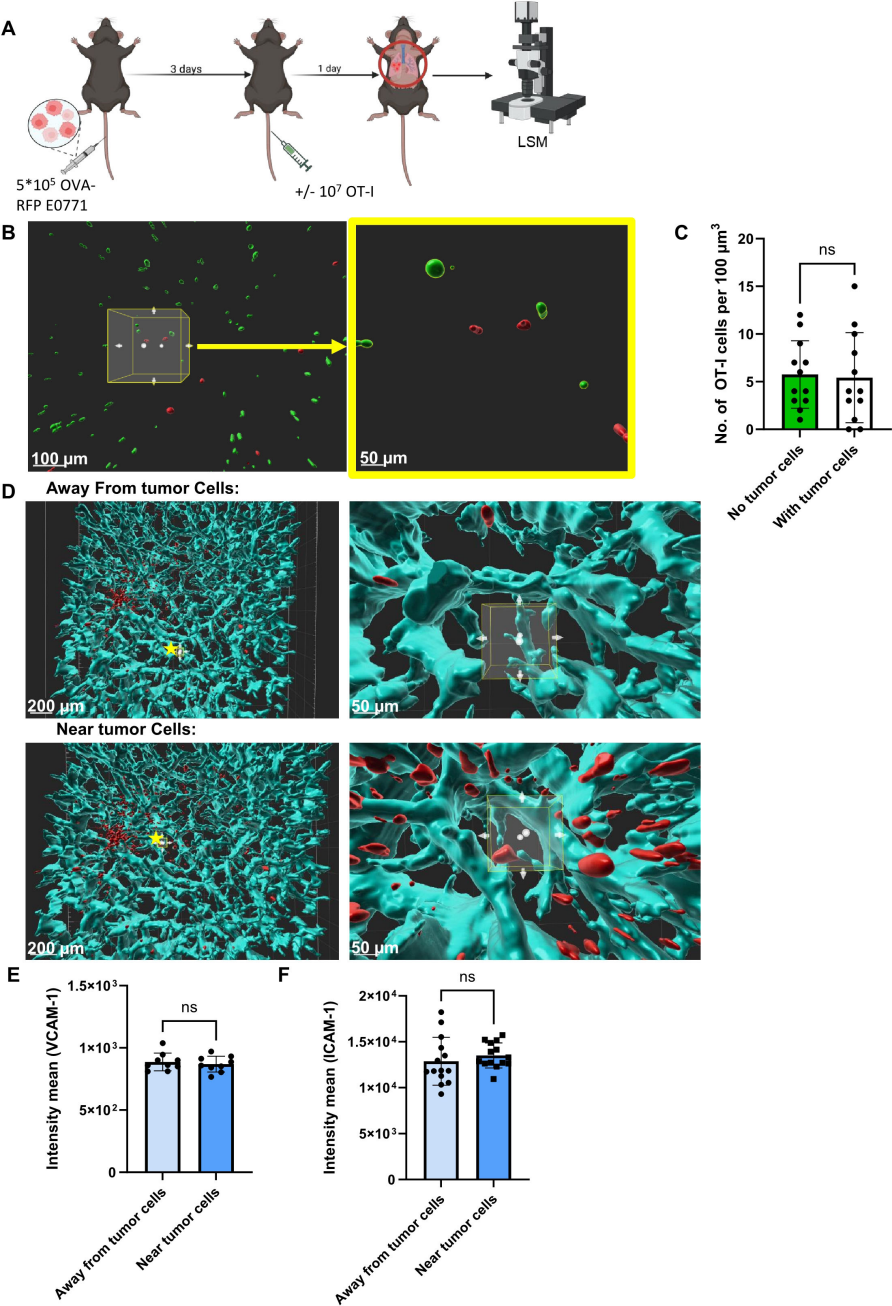
Spatial distribution of Ag specific CTLs and lung residing E0771 breast cancer cells. **(A)** A scheme depicting the experimental procedure. 5x10^5^ OVA-RFP E0771 cells were i.v. injected to recipient mice. 3 days later 10^7^ CFSE-labeled OT-I CTLs were injected to the same mice. One day post CTL injection, lungs were harvested and processed for LSM. **(B)** LSM images of OVA-RFP E0771 and CFSE-stained OT-I CTLs. Right panel- enlargement of the cube in the left panel. Left bar, 100 µm; right bar, 50 µm. **(C)** Average number of OT-I CTLs inside a lung area occupied by E0771 tumors cells, as compared to identical randomly selected area of tumor-free lung compartment. The average OT-I CTL densities in 5 tumor-free lung cubic volumes were compared to averaged OT-I CTL densities in 30 tumor-containing lung cubic volumes. **(D)** 100 µm^3^ cubes were randomly chosen for analysis of VCAM-1 density (cyan) either nearby or remote from the E0771 cells (red). Right panels- enlargements of the starred cubes depicted in the left panels. Left bars, 100 µm; Right bars, 50 µm. **(E)** Mean intensity of VCAM-1 near E0771 cells within a distance smaller than 100 micron or remote from the cancer cells. n=9 for either cell containing or cell free lung cubes. **(F)** Mean intensity of ICAM-1 nearby or remote from E0771 cells determined as in **(B)**. n=14 for either cell-containing or cell-free lung cubes.

To follow if identically introduced OT-I CTLs can also encounter and kill OVA-RFP E0771 cells which have reached the lungs, we next introduced these tumor cells to the lungs in an experimental metastasis model. Combined with intravenous injection of cognate OT-I CTLs, we could follow the fate of the metastatic E0771 cells shortly after they resided in the lungs by FACS analysis of total lung cell suspensions (**Figure 2A**). Strikingly, OT-I CTLs introduced intravenously 3 days after accumulation of OVA-RFP E0771 cells in the lungs failed to eliminate the metastatic cells inside the lungs as evaluated by FACS (**Figure 2B**). Since FACS analysis is based on harvesting metastatic cells from total lung cell suspension, a standard method that does not recover all lung entrapped cells, we decided to use a direct 3D imaging of whole lipid cleared lung lobes harvested from similar experiments and quantify the number of OVA-RFP E0771 cells residing in lungs subjected to intravenously introduced OT-I CTLs. We first verified that the OVA-RFP E0771 cells accumulated in the lungs were readily visible by 3D LSM-based imaging of whole lungs cleared of their lipids (**Supplementary Figure 5**). The recipient mice were next introduced with OVA-RFP E0771 and subjected to intravital staining with an Alexa-647 labeled mAb to the pan endothelial marker CD31 before lung harvesting and processing for LSM analysis. This approach allowed us to determine both the number of OVA-RFP E0771 cells remaining in the lungs and their partition between intra and extravascular lung compartments. This approach indicated that nearly all viable OVA-RFP E0771 cells resided inside pulmonary blood vessels (**Supplementary Figure 6**; **Supplementary Video 1**). Importantly, and in support of the FACS analysis of total lung cell suspensions, none of OVA-RFP E0771 cells residing in the lungs were eliminated by OT-I CTLs (**Figure 2C**). These results collectively suggest that while E0771 breast cancer cells that express a potent neoantigen like OVA are susceptible to cognate OT-I CTLs reaching the mammary fat pad, these cells, once residing inside the lung vasculature, are fully protected from *in vivo* killing by similarly intravenously introduced OT-I CTLs. We next applied 3D LSM based lung imaging to follow if intravenously introduced OT-I CTLs passing through lungs, in which OVA RFP expressing E0771 had accumulated, were actively recruited by the lung residing breast cancer cells. We injected OVA-expressing tumor cells into the mice and 3 days later introduced CFSE labeled OT-I CTLs, harvested the lungs 24 hours later, and subjected them to processing and clearance followed by LSM based 3D analysis (**Figures 3A, B**). Surprisingly, the density of OT-I CTLs accumulated nearby OVA E0771 cells residing in the lungs was identical to that of CTLs accumulated far away from sites occupied by the E0771 cells residing inside lung blood vessels (**Figures 3B, C**; **Supplementary Video 2**). Furthermore, OVA-RFP E0771 cells surviving inside the lung vasculature failed to locally elevate the relative vascular expression levels of two main vascular adhesion ligands readily recognized by CTLs, VCAM-1 and ICAM-1 (**Figures 3D–F**). These results collectively indicated that OVA RFP expressing E0771 cells residing inside the lung vasculature do not actively recruit OT-I CTLs to their vicinity. Thus, systemic circulating CTLs randomly pass through the lung microvasculature without encountering recruitment signals from metastatic E0771 cells residing inside lung blood vessels.

### OVA and RFP co-expressing E0771 cells evade killing by OT-I CTLs in a spontaneous model of breast cancer metastasis to lungs

**Figure 4:**
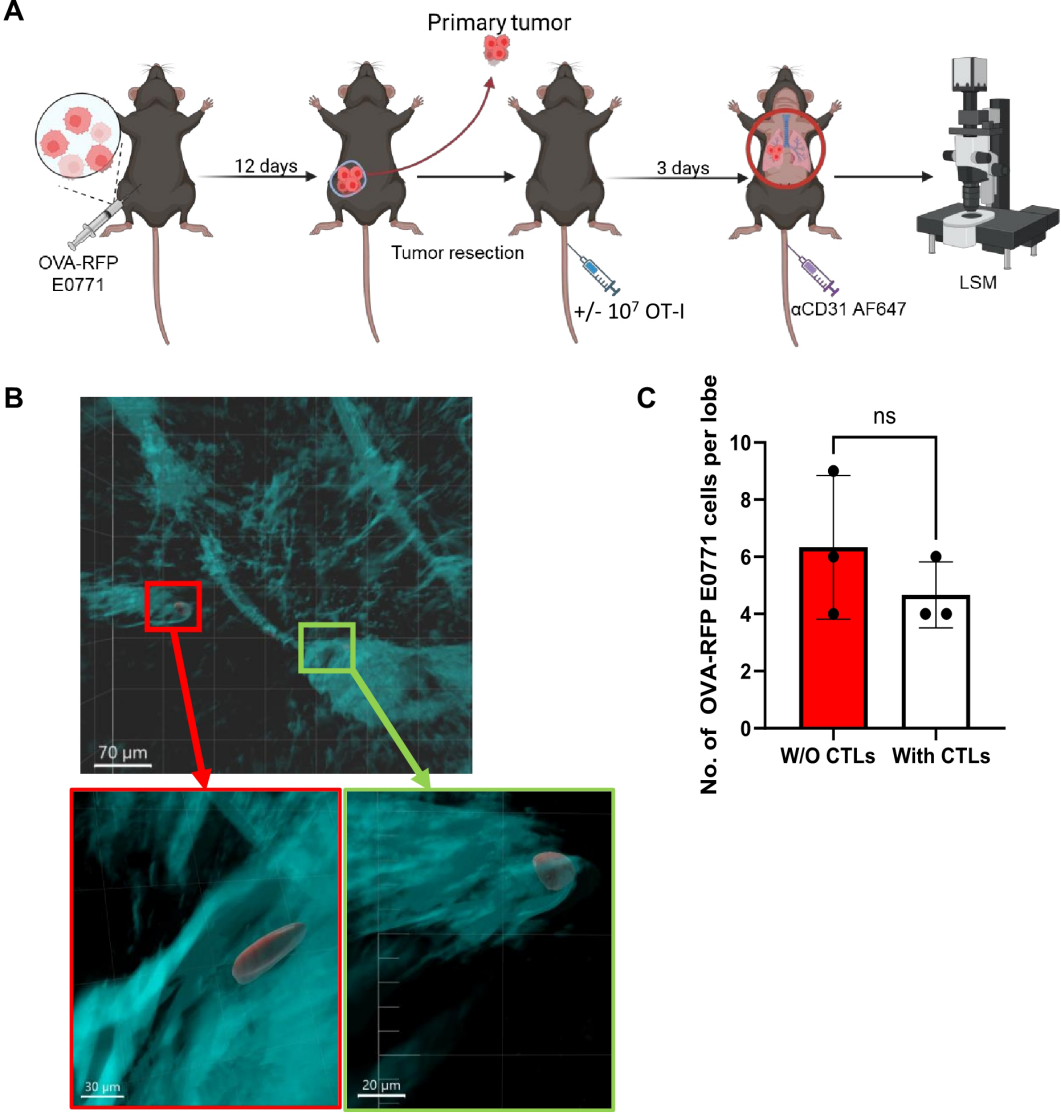
OVA-specific CTLs fail to kill OVA-RFP-E0771 cells in a spontaneous lung metastasis model. **(A)** Scheme of the spontaneous metastasis model: 3x10^5^ OVA-RFP-E0771 cells were implanted into the mammary fat pad of female C57BL/6 mice and 12 days later primary tumors were resected. Mice were left intact or injected with OT-I CTLs. 3 days later Alexa-647 labeled anti-CD31 was injected i.v. and 5 mins later mice were sacrificed, and lungs were harvested and processed for LSM analysis. **(B)** A representative LSM image of spontaneous metastasis of E0771 cells in the lungs as described in **(A)**. Red, OVA-RFP-E0771 cells, Cyan, CD31-positive vessels. Red and green squares are magnified in the panels below. Top bar, 70 µm; bottom left bar, 30 µm; bottom right bar, 20 µm. **(C)** The effect of i.v. injected OT-I CTLs on the number of OVA-RFP-E0771 cells remaining in the lungs. For each experimental condition 3 lobes from 2 spontaneous metastasis bearing mice were analyzed. The scatter plots and bars depict the mean values ± SEM. ns= non-significant.

To further test the susceptibility of OVA expressing RFP E0771 cells to killing by OT-I T cells in a more physiological setting, we next analyzed spontaneous breast cancer metastasis to lungs. Notably, in this model, the expression of ICAM-1 on E0771 cells drives a neutrophil mediated anti-metastatic response, resulting in low rates of survival of these breast cancer cells in the lungs (29). Thus, FACS based enumeration of the number of OVA-expressing RFP E0771 remaining after injection of OT-I T cells was essentially impossible. Direct visualization of the metastatic breast cancer cells in the lungs was therefore necessary to overcome the limited ability to achieve complete cell separation and harvesting of residual metastatic E0771 cells in enzyme treated lung samples. Using LSM microscopy of whole lungs, we successfully detected OVA expressing RFP labeled E0771 reaching the lungs after resection of primary OVA-RFP E0771 tumors and determined their partition between intravascular and extravascular lung compartments. Notably, all metastatic OVA RFP E0771 cells accumulated preferentially inside the lung vasculature under different settings of spontaneous lung metastasis (**Figures 4A, B**). Consistent with the experimental metastasis results, intravenously introduced OT-I CTLs also failed to kill lung residing OVA-RFP E0771 cells disseminated from primary breast tumors following resection (**Figure 4C**). This failure was particularly surprising given that all OVA-RFP E0771 resided inside lung blood vessels. Thus, in both experimental and spontaneous lung metastasis models of OVA expressing E0771 cells, these breast cancer cells were fully protected from OVA-specific CTL mediated killing under distinct conditions and at different time windows.

### OT-I CTLs reaching the lungs kill OVA-expressing melanoma cells

**Figure 5:**
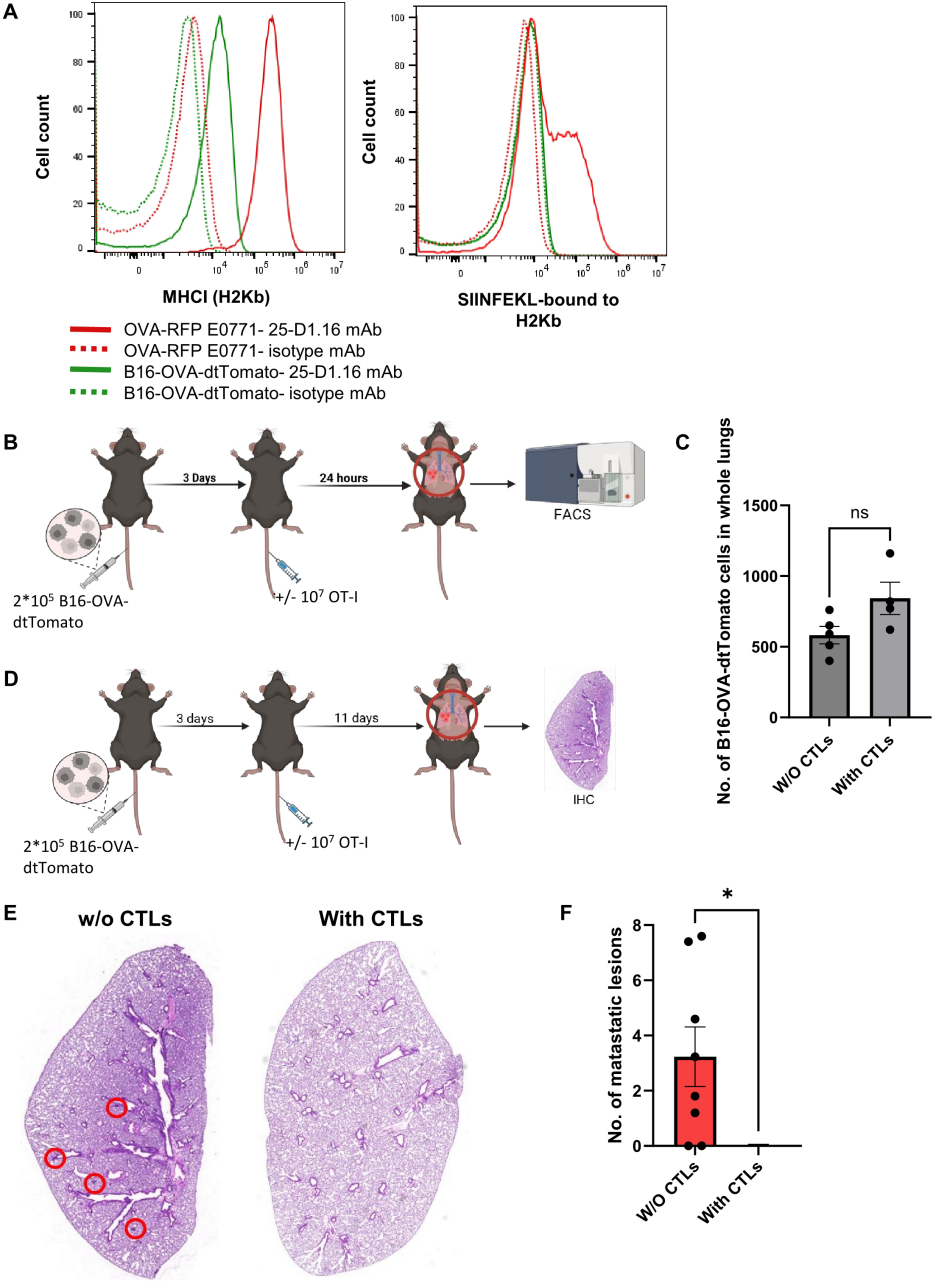
Lung metastases of OVA-expressing B16 melanoma are eliminated with time by intravenously transferred OT-I CTLs. **(A)**The histograms in the left panel depict the H2Kb MHC-I surface levels of each of the cell lines. Control staining of each cell line with isotype matched mAb is shown in the dotted plots. The right panel depicts the surface levels of the SIINFEKL-H2Kb complex on each of the cell lines detected with the pMHC specific 25-D1.16 mAb. **(B, C)** 2x10^5^ B16-OVA-tomato cells were intravenously injected into mice, and 3 days later 10^7^ OT-I T-cells were injected into one of the mice groups while another group was left intact. 24 hours post T cell injection, lungs were harvested and the numbers of B16-OVA-dtTomato cells recovered from total lung cell suspensions were determined by FACS. **(D)** 2x10^5^ B16-OVA-dtTomato cells were intravenously injected into mice, and 3 days later 10^7^ OT-I T-cells were injected into one of the mice groups. Two weeks post tumor cell injection, lungs were harvested and processed for immunohistochemistry. **(E)** Representative paraffin section images of lungs containing OVA B16 lesions following sham (left) vs OT-I CTL injection (right). Metastatic lesions are marked by red circles. **(F)** The number of B16 metastatic lesions detected in histological lung sections determined in E. n=7 sections collected from 3 mice of each experimental group. The scatter plots depict the mean values ± SEM. *p<0.05.

The immune evasion of our OVA-RFP E0771 breast cancer cells from CTL mediated killing in the lungs could be due to unexpected lung specific OT-I CTL exhaustion. We, therefore, tested the ability of identically generated intravenously injected OT-I CTLs to eliminate tomato-OVA B16 cells, a widely used model of melanoma metastasis to lungs (48). These cells express negligible levels of MHC-I and hence, we could not detect SIINFEKL-H-2Kb complexes on the surface of these cells before and after their entering the lung parenchyma (**Figure 5A**). We introduced these tumor cells to the lungs in an experimental metastasis model followed by intravenous injection of cognate OT-I CTLs 3 days later and followed the fate of the metastatic melanoma cells before and after introduction of OT-I CTLs. Notably, the OVA-B16 cells residing in the lungs did not undergo killing by the CTLs within the first 24 hrs after injection (**Figures 5B, C**). Nevertheless, and in sharp contrast to OVA-expressing E0771 cells, the OVA-expressing B16 melanoma cells were later eliminated by intravenously injected OT-I CTLs (**Figures 5D–F**). Importantly, these results indicated, that OT-I CTLs reaching the lungs are not intrinsically exhausted and that their failure to eliminate the lung residing OVA-E0771 cells resides in a lung-specific evasion of the OVA-expressing breast cancer cells from CTL mediated killing. Since circulating tumor cells (CTCs) reaching the lungs are often coated with platelets and thereby get protected from both NK and CTL mediated killing (49), we next compared the mean platelet coating of either OVA expressing B16 cells or OVA-expressing RFP E0771 accumulating in the lungs soon after intravenous injection. Interestingly, both intravenously injected E0771 and OVA-expressing B16 were similarly coated with CD41 platelets (**Supplementary Figure 7**). Thus, the failure of OT-I CTLs to kill OVA-expressing E0771 could not be attributed to immune escape resulting of higher platelet coating of these cells inside the lungs.

### OVA RFP expressing E0771 cells rapidly lose OVA-derived SIINFEKL peptide pMHC expression in the lungs but retain this pMHC in primary breast tumors

**Figure 6:**
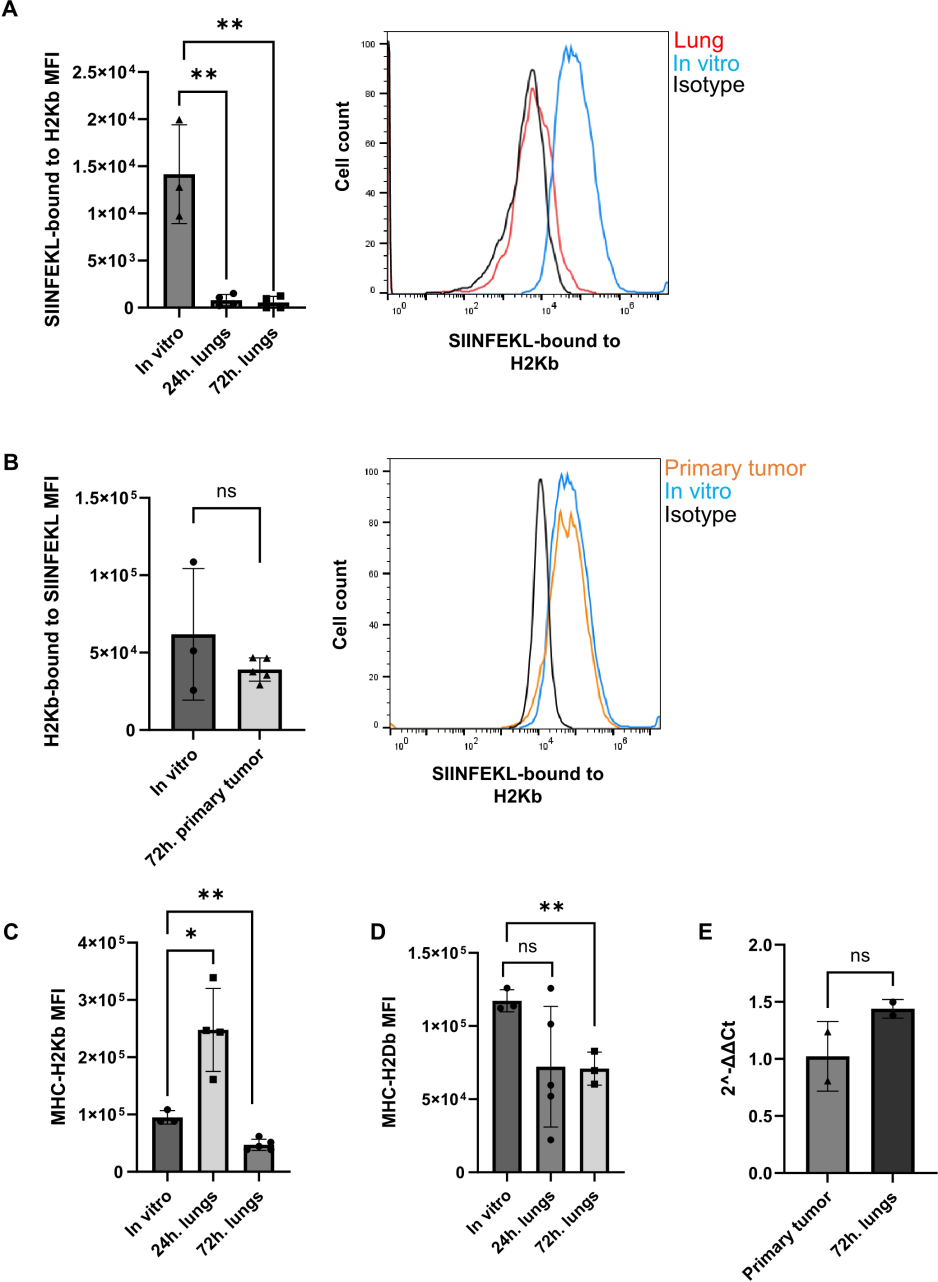
Abolished presentation of MHC-I-OVA peptide in OVA expressing E0771 cells reaching the lungs but not the primary tumor tissue. **(A)** Left panel: The mean fluorescence intensity (MFI) of the SIINFEKL-H2-Kb pMHC epitope recognized by the 25-D1.16 mAb, averaged for the entire OVA-RFP E0771 populations harvested from lungs either 24 hrs. or 72 hrs. post i.v. injection. The MFI values were averaged for 3 independent experiments. The scatter plots and bars depict the mean values ± SEM. **p<0.001. Right panel: The surface levels of SIINFEKL-H2-Kb pMHC complexes on OVA-RFP E0771 recovered from recipient lungs 24 hrs. after i.v. injection (red line). The level of this pMHC on the originally injected OVA-RFP E0771 cells is depicted by the blue histogram. Background staining of OVA-RFP E0771 with isotype matched control mAb is depicted by the black histogram. **(B)** Left panel: The scatter plots and bars in the inset depict the averaged MFI values ± SEM of 2 samples of originally implanted OVA-RFP E0771 cells and 5 samples of OVA-RFP E0771 cells recovered from the primary tumor sites on day 3 post implantation. ns= non-significant. Right panel: The surface levels of SIINFEKL-H2-Kb pMHC on OVA-RFP E0771 recovered from recipient mammary fat pad 3 days after cell implantation (orange histogram). The pMHC signal detected on the originally implanted OVA-RFP E0771 cells is depicted by the blue histogram. Background staining of OVA-RFP E0771 with isotype matched control mAb is depicted by the black histogram. **(C)** The levels of H2-Kb MHC-I on originally injected OVA-RFP E0771 cells as compared to OVA-RFP E0771 recovered from lungs 24 and 72 hrs. post i.v. injection. The scatter plots and bars depict the mean values ± SEM. *p<0.05. n= 4. **(D)** The levels of H2-Db MHC-I on originally injected OVA-RFP E0771 cells as compared to E0771-OVA-RFP recovered from lungs 72 hrs. post injection. The scatter plots and bars depict the mean values ± SEM. *p<0.05. n= 4. **(E)** qPCR of MHCI-H2Kb transcription in OVA-RFP E0771 recovered from either primary tumors on day 3 post implantation or from lungs 72 hrs post i.v. injection. The scatter plots and bars depict the mean values of relative fold change in gene expression (2^-ΔΔCt^) ± SEM. *p<0.05. n= 2.

The inability of circulating OT-I CTLs to kill the OVA-expressing E0771 cells residing in the lung vasculature could be the result of poor recruitment by these metastatic cells. Another possibility for the poor killing could be inefficient CTL recognition of the OT-I specific OVA peptide pMHC complex on the surface of the breast cancer cells residing inside the lung vasculature. We therefore compared the expression levels of the SIINFEKL-H-2Kb pMHC-I complex on OVA-RFP E0771 cells accumulating inside the lungs 1–3 days after intravenous injection. Strikingly, all OVA-RFP E0771 cells lost expression of SIINFEKL-H-2Kb pMHC within the first 24 hrs of their entry into the lung vasculature (**Figure 6A**) and failed to regain expression of this pMHC 72 hrs after their initial injection (**Figure 6A**). In contrast, and in agreement with their high susceptibility to killing by intravenously injected OT-I CTLs, OVA RFP expressing E0771 cells implanted in the mammary fat pad retained their high SIINFEKL- H-2Kb pMHC expression (**Figure 6B**). Interestingly, OVA-RFP E0771 cells retained their surface H-2Kb as well as H-2Db early after entry to the lung vasculature (**Figures 6C, D**). Furthermore H-2Kb mRNA levels were also retained in the lung-accumulating OVA-RFP E0771 during the first 72 hours after i.v. injection (**Figure 6E**). In contrast, 72 hours after initial entry to the lung vasculature, the surface levels of both H-2Kb and H-2Db significantly declined, consistent with impaired loading of self-peptides on both MHC-I molecules (**Figures 6C, D**). These results collectively suggested that OVA-RFP E0771 cells lost their OVA neoantigen-derived peptide/MHC complex, as well as other pMHC complexes from their cell surface due to a lung-specific interference with their peptide MHC-I presentation machinery.

**Figure 7:**
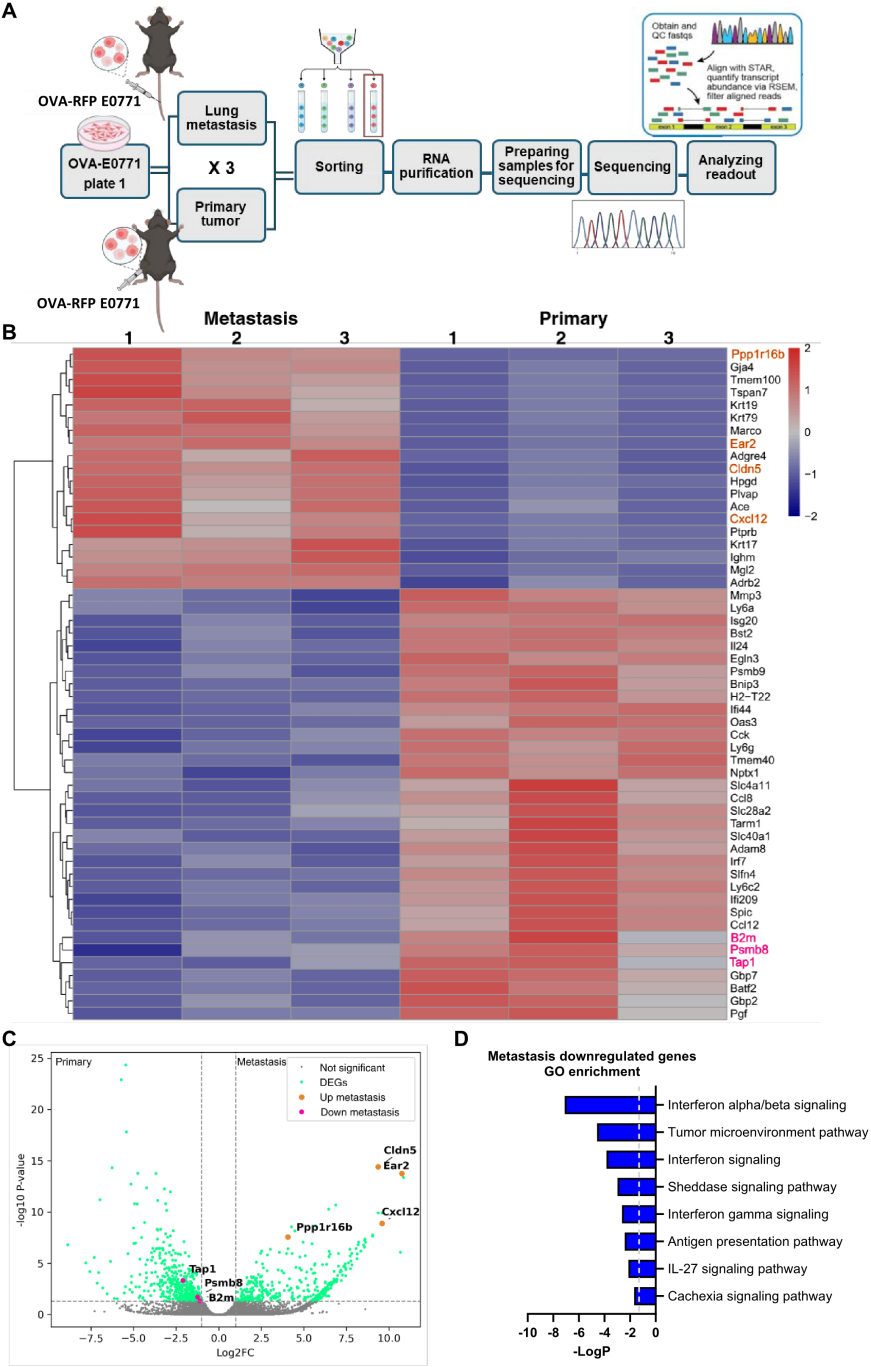
Transcriptomic analysis of primary tumor and lung residing OVA-E0771 cells. **(A)** A scheme depicting the experimental procedure. OVA-RFP-E0771 cells were isolated and sorted from primary tumors or lungs at the indicated time points. RNA was extracted and sequenced as described in the methods section. Differential expression analysis was performed using DESeq2 as described in the methods section. **(B)** A heatmap of representative differentially expressed genes (DEG) for the compared groups as described in **(A)**. Each group consisted of 1500 E0771 cells isolated from primary tumors or lungs. (n =3 mice). Right list: Pink, downregulated genes of interest; orange, representative upregulated genes. Legend on the top right side, z-score. **(C)** Volcano plot of the differentially expressed genes (DEG). Pink dots, downregulated genes of interest; orange dots, upregulated genes of interest; green dots- differentially expressed genes; grey dots, genes not meeting the significance threshold, padj ≤ 0.05. **(D)** Main pathways downregulated in lung-residing E0771 cells, compared to the primary tumor. Pathway analysis was done using IPA, top 8 downregulated pathways were chosen for representation.

To further dissect the mechanisms by which OVA-RFP E0771 cells evade killing by OT-I CTLs entering the lungs, we next performed bulk RNA-seq analysis of either E0771 cells implanted inside primary tumors or reaching and residing in the lung vasculature (**Figure 7A**). Bioinformatic analysis indicated that 313 genes were upregulated and 290 were downregulated in lung-residing OVA-RFP E0771 cells compared with their counterparts isolated from the primary tumors (**Figures 7B–D**). Among these genes, three major effectors of MHC-I restricted antigen presentation were downregulated: B2M (Beta-2-Microglobulin) (50); TAP1, a key component of the antigen processing and presentation pathway (TAP1/TAP2) (51, 52); PSMB8 (Proteasome Subunit Beta 8) a β5i subunit of the immunoproteasome also involved in protein processing for MHC-I presentation (53, 54). In contrast, OVA transcription in lung residing E0771 cells did not decrease. Notably, E0771 cells residing in the lung vasculature dramatically upregulated the chemokine CXCL12 (log2FC= 9.59, padj<0.0001). This chemokine might contribute to autocrine CXCR4-dependent survival of breast cancer cells (55–57). Pathway analysis also suggested that the lung residing OVA-RFP E0771 cells were less responsive to IFNβ signaling (**Figure 7D**), further accounting for their reduced surface pMHC-I levels. Interestingly, however, E0771 cells recovered from lungs transcribed much higher levels of IFN-γ receptor type 1 (IFNGR1) than in the primary tumor site (log2FC= 1.91, padj< 0.00061). Taken together, these results suggest that the loss of pMHC presentation on E0771 cells residing inside lung blood vessels is likely a result of the downregulation of multiple genes involved in neoantigen processing and presentation mediated by IFN-related pathways.

### Expression of pMHC by transient SIINFEKL peptide loading is insufficient for OT-I CTL mediated recruitment of killing

**Figure 8:**
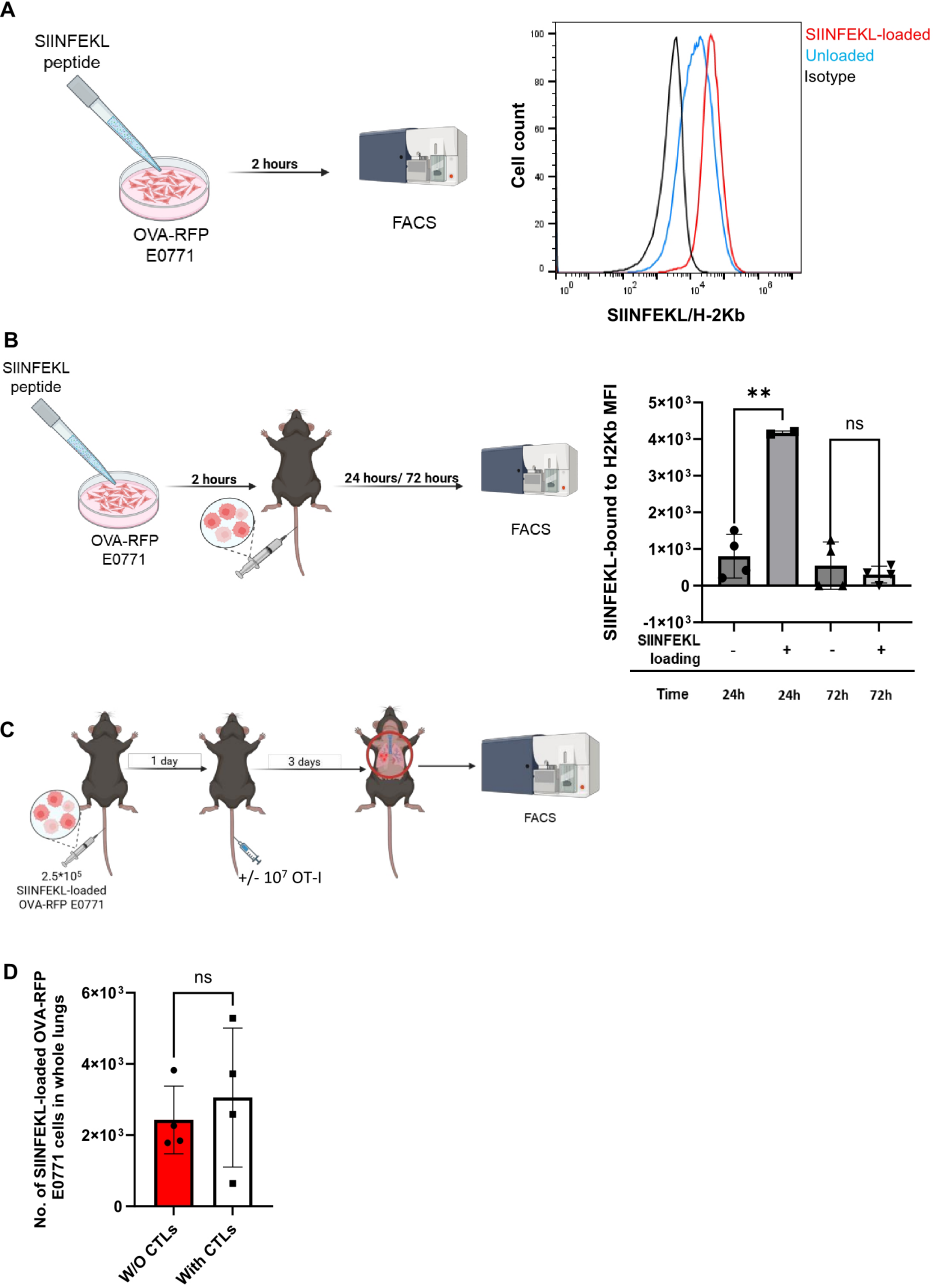
Exogenous SIINFEKL-MHC-I presentation is insufficient to override the escape of lung populating E0771 cells from OT-I CTL killing. **(A)** OVA-RFP E0771 cells were incubated with saturating levels (2µg/ml) of SIINFEKL peptide for 2 hours. The surface levels of SIINFEKL-H2-Kb detected with the 25-D1.16 mAb are shown for isotype matched control (black), unloaded (blue line) and for SIINFEKL peptide loaded OVA-RFP E0771 cells analyzed *in vitro* (red line). **(B)** The SIINFEKL-peptide loaded OVA-RFP E0771 cells analyzed in A were injected i.v. to recipient mice, cells were isolated 24 and 72 hrs later from total lung suspensions of recipient mice, and their surface levels of SIINFEKL-H2-Kb complex were determined by staining with the 25-D1.16 mAb. Note that SIINFEKL-H2Kb presentation remains high 24 hrs after E0771 injection while 72 hrs later, the loaded peptide is no longer presented by the lung populating E0771 cells. n= 4. The scatter plots and bars depict the mean values ± SEM. **p<0.001. **(C)** A scheme of the CTL mediated killing experiment. 2.5 x 10^5^ SIINFEKL-loaded OVA-RFP E0771 cells were i.v. injected to the mice. 24 hours later 10^7^ OT-I CTLs were injected to the mice. 3 days post T-cell injection, mice were sacrificed and the number of OVA-RFP E0771 cells was analyzed in total lung cell suspensions. **(D)** Effect of i.v. injected OT-I CTL on the survival of SIINFEKL loaded OVA-RFP E0771 in the lungs quantified as described in **(C)**. The results are the mean ± SEM of four experiments. ns, non-significant.

H-2Kb expressing cells can bind with high affinity exogenously introduced OVA derived SIINFEKL peptide (58). We therefore attempted to assess if a temporal loading of SIINFEKL-H-2Kb pMHC expression in lung-populating OVA-RFP E0771 cells by *in vitro* loading with SIINFEKL peptide could promote their *in vivo* killing by OT-I CTLs. *In vitro* preloading of the OVA-RFP E0771 cells with a saturating dose of the SIINFEKL peptide prior to their injection into recipient mice (**Figure 8A**) resulted in a significant retention of SIINFEKL-H-2Kb pMHC complexes on the surface of the cells accumulating in the lungs 24 hrs. after their intravenous injection into the recipient mice (**Figure 8B**, second bar). As expected, 72 hrs after injection of the peptide-loaded E0771 cells, SIINFEKL-H-2Kb pMHC complex expression on lung populating E0771 cells was lost (**Figure 8B**, 4^th^ bar). We could therefore assess the killing potential of OT-I CTLs towards the SIINFEKL loaded OVA-RFP E0771 cells only within the first 24 hrs after their accumulation in the lungs. Interestingly, intravenously injected OT-I CTLs reaching the lungs within that time window could not eliminate the SIINFEKL loaded lung-residing OVA-RFP E0771 cells (**Figures 8C, D**). These findings indicated that a transient presence of the OT-I binding pMHC complex on E0771 cells residing inside the lung vasculature at the time point of cognate OT-I CTL accumulation inside the lungs was insufficient for their effective killing by these CTLs. The forced pMHC presentation on the surface of E0771 cancer cells residing in the lungs was also insufficient for OT-I CTL enrichment inside intravascular sites occupied by lung resident SIINFEKL loaded OVA-RFP E0771, as was confirmed by LSM analysis (**Supplementary Figures 8A–C**).

### Bypassing OVA processing through stable SIINFEKL expression rescues the escape of EO771 cells from CTL killing in the lungs

**Figure 9:**
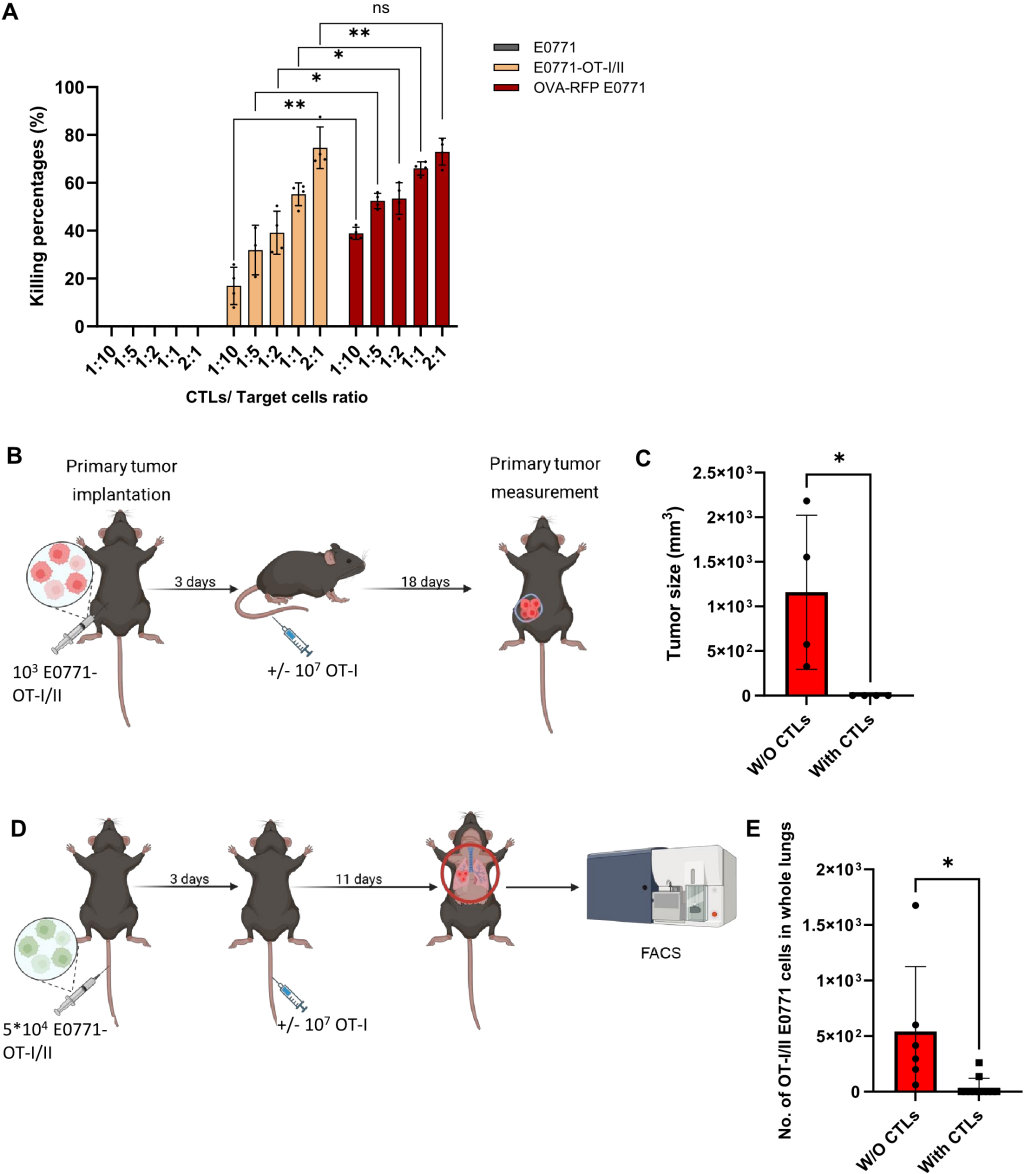
E0771 cells expressing OVA derived OT-I peptide are killed by OT-I CTLs both in primary tumors and in the lungs. **(A)***In vitro* killing of OT-I/II peptide expressing E0771 cells by OT-I CTLs. Either OT-I/II peptide expressing or OVA RFP expressing E0771 cells were cultured in a 96 well plate O.N. CSFE labeled OT-I CTLs were settled on the different cancer cells at the indicated CTL to target ratios. One day later, the number of viable E0771 cancer cells remaining in each well was determined by FACS. n= 4 mice. The scatter plots and bars depict the mean values ± SEM. *p<0.05. **p<0.001. **(B)** A scheme depicting the experimental procedure. *In vivo* killing of primary breast tumors consisting of OT-I/II peptide expressing E0771 by OT-I CTLs. WT mice were implanted with 1,000 tumor cells into the mammary fat pad, and 3 days later OT-I T cells were i.v. injected into the tumor bearing mice. Mice were sacrificed 18 days after CTL injection and tumor size was determined by weight. **(C)**. Results of the experiment described in **(B)**. n= 4 mice. The scatter plots and bars depict the mean values ± SEM. *p<0.05. **(D)** Effect of i.v. injected OT-I CTL on the survival of OT-I/II peptide expressing E0771 cells in the lungs. A scheme depicting the experimental procedure. 5x 10^4^ OT-I/II peptide E0771 cells were injected into recipient mice and 3 days later 10^7^ OT-I CTLs were injected i.v. Control mice injected with GFP OT-I/II peptide E0771 cells were left untreated. 11 days later mice were euthanized, lungs were harvested, and the number of viable OT-I/II E0771 in total lung cell suspensions were determined by FACS analysis. Without CTLs, n=6 mice; with CTLs, n=11 mice. **(E)** The results are the mean ± SEM of four experiments. The scatter plots and bars depict the mean values ± SEM. *p<0.05.

To further test whether the lung specific loss of OVA-derived SIINFEKL peptide presentation on E0771 cells is indeed due to defective OVA processing, we generated a new E0771 variant (E0771 OT-I/II) introduced with a vector encoding a short SIINFEKL peptide which can load on the MHC-I H-2Kb independently of OVA processing (**Supplementary Figure 1**). As expected, this variant E0771 line was selectively killed *in vitro* by OT-I T cells (**Figure 9A**) and was readily eliminated by intravenously injected OT-I CTLs when implanted inside mammary fat pads (**Figures 9B, C**). We could not confirm, however, by FACS that the cells retained their original SIINFEKL-H-2Kb presentation because SIINFEKL-H-2Kb expression was below the detection limit (**Supplementary Figure 1**). Notably, upon entering the lungs, this E0771 variant was eliminated by OT-I CTLs (**Figures 9D, E**) which were identically injected intravenously as in previous parts. This result suggests that direct and stable presentation of the OVA SIINFEKL peptide on E0771 bypassing the OVA processing machineries renders these cells susceptible to recognition and killing by lung circulating OT-I CTLs. Taken together, we conclude that OT-I mediated killing of metastatic E0771 cells in the lungs requires persistent expression of MHC-I-SIINFEKL complexes on the surface of these breast cancer cells as they reside in the lungs.

## Discussion

Adoptive cell transfer (ACT) of tumor-killer immune cells (TILs, CAR-T, CAR-NK cells and other engineered leukocytes) (9, 10) has become a major therapeutic tool in immunotherapy (59), in addition to immune checkpoint blockade therapy (60). The use of tumor-specific CTLs injected systemically after *in vitro* activation has become a major line of immunotherapy for eradication of residual primary tumors (61, 62). However, the application of TIL-based therapy for clearing residual metastatic lesions arising from a given tumor is still questionable. Whether tumor-specific TILs can be effectively directed to target micro-metastases that develop very early in the multi-step metastatic process is a question of keen clinical significance.

In the present work, we have tried to address this standing question in two prototypic murine models of breast and skin cancer metastasis, using a monoclonal CTL specific for a neoantigen ectopically expressed in the two cancer types. We followed the response of two OVA-expressing tumor cells, namely the breast cancer line E0771 and the melanoma cancer line B16, introduced into immunocompetent C57BL/6 recipient mice to intravenously injected CTLs derived from the CD8 T cell OT-I transgene (63–66). We first confirmed the potent *in vivo* killing activity of intravenously injected OT-I CTLs towards primary OVA-expressing E0771 breast tumors orthotopically implanted in mammary fat pads. Surprisingly, the same OT-I CTLs, although continuously circulating through the lung vasculature, failed to encounter and eliminate OVA-expressing E0771 cells residing in the lungs. Nevertheless, the same identically intravenously introduced OT-I CTLs readily eliminated OVA-expressing B16 melanoma cells accumulating in the lungs even though OVA B16 cells expressed much lower levels of OT-I specific SIINFEKL-H2Kb pMHC complexes than the OVA-expressing E0771 cells. When systematically dissecting the location of both E0771 in distinct lung compartments and of OT-I CTLs passing through the lung vasculature, we found in multiple experimental models that OVA expressing E0771 survived inside lung blood vessels rather than in extravascular lung compartments, consistent with our previous findings on parental E0771 (29). These findings were also confirmed by a recent work which reported that endothelium-derived angiocrine Wnt signals allow these cells to survive and proliferate inside the lung vasculature (67). Counterintuitively, these intravascular OVA-expressing E0771 cells were completely ignored by OT-I CTLs passing through the lung vasculature. Our results indicate that a subset of metastatic cells entrapped inside lung vessels escape CTL mediated killing primarily by a rapid lung specific loss of MHC-I restricted neoantigen presentation.

The dramatic loss of OVA derived SIINFEKL-H2Kb pMHC expression on the surface of the lung residing OVA expressing E0771 cells sharply contrasted the normal pMHC expression retained by these cells in primary tumors and their high killing by intravenously injected adoptively transferred OT-I CTLs. What could be the molecular basis of the rapid pMHC loss on E0771 cells and its apparent restriction to the lung? Our work suggests that while the pMHC complex recognized by the OT-I TCR (SIINFEKL-H2Kb) was diminished during the first 24–48 hours after initial E0771 accumulation inside the lung vasculature, the H2Kb MHC-I protein itself was initially retained but slowly declined later on, reflecting a major impairment in OVA processing and the presentation of OVA-derived H2Kb associated peptides on lung residing E0771 cells. Intriguingly, this slow decline in MHC-I H2Kb expression by lung residing OVA E0771 cells was not the result of downregulated MHC-I transcription, further suggesting that it is the specific antigen presentation machinery critical for protein processing and assembly of peptide bound MHC-I complexes on the surface of E0771 cells which is primarily impaired in E0771 cells residing in the lungs. Consistent with this postulated escape mechanism, an E0771 variant engineered to express the SIINFEKL peptide rather than the entire OVA antigen was efficiently recognized and killed by OT-I CTLs entering the lungs. To identify MHC restricted antigen presentation factors selectively downregulated in E0771 reaching the lungs, but retained in E0771 residing inside mammary fat pads, we performed bulk RNA-seq analysis of OVA-expressing E0771 cells isolated from either primary tumors or lungs. Indeed, our transcriptomic analysis identified a significant downregulation of B2M, TAP1 and PSMB8 (50–52, 54) involved in antigen processing and presentation on MHC-I complexes. Our transcriptomic analysis also suggested that the CXCL12 chemokine, found to be dramatically upregulated in E0771 residing inside lung vessels (775-fold), might provide autocrine pro-survival signals to these breast cancer cells inside the lung vasculature.

Our results are a first indication that a subset of breast cancer metastatic cells reaching the lungs escape killing by tumor neoantigen specific CTLs via a loss of neopeptide expression. This lung specific escape appears to be the result of a defective antigen processing machinery operating downstream of IFN dependent responses. This loss could reflect a lung specific starvation of these cells for IFN-γ, a potent regulator of antigen processing and MHC-I restricted antigen presentation (68). Whether lungs contain reduced levels of this cytokine compared to primary breast tumors is unknown. Interestingly, E0771 cells residing inside the lung vasculature retained IFN-γR transcription. Notably, the lung specific MHC-I related tumor escape mechanism identified in the present study was triggered much more rapidly than classic cancer evasion mechanisms that evolve over weeks to years in experimental animals and humans and involves immune-editing of singular clones (69). An open question is whether the residence of E0771 cells inside intravascular lung niches is the cause of their escape from CTL encounter and killing. As our studies are based on a few cancer cell lines, at present, our findings cannot be extrapolated to other cancer subsets. Future studies with additional cell lines in different mice strains are required to further extend our new findings.

## Data Availability

The data discussed in this publication have been deposited in NCBI's Gene Expression Omnibus (Edgar et al., 2002) and are accessible through GEO Series accession number GSE316228 (https://www.ncbi.nlm.nih.gov/geo/query/acc.cgi?acc=GSE316228).
