# Supplementary material for: A lung specific escape of intravascular metastatic breast cancer cells from cytotoxic T cell killing
Source: Front Immunol. 2026 Jan 22;16:1599751. doi: 10.3389/fimmu.2025.1599751 (PMC12872491; doi:10.3389/fimmu.2025.1599751)
Supplement: Supplementary file 1 [file Presentation1.pptx]

## Slide 1
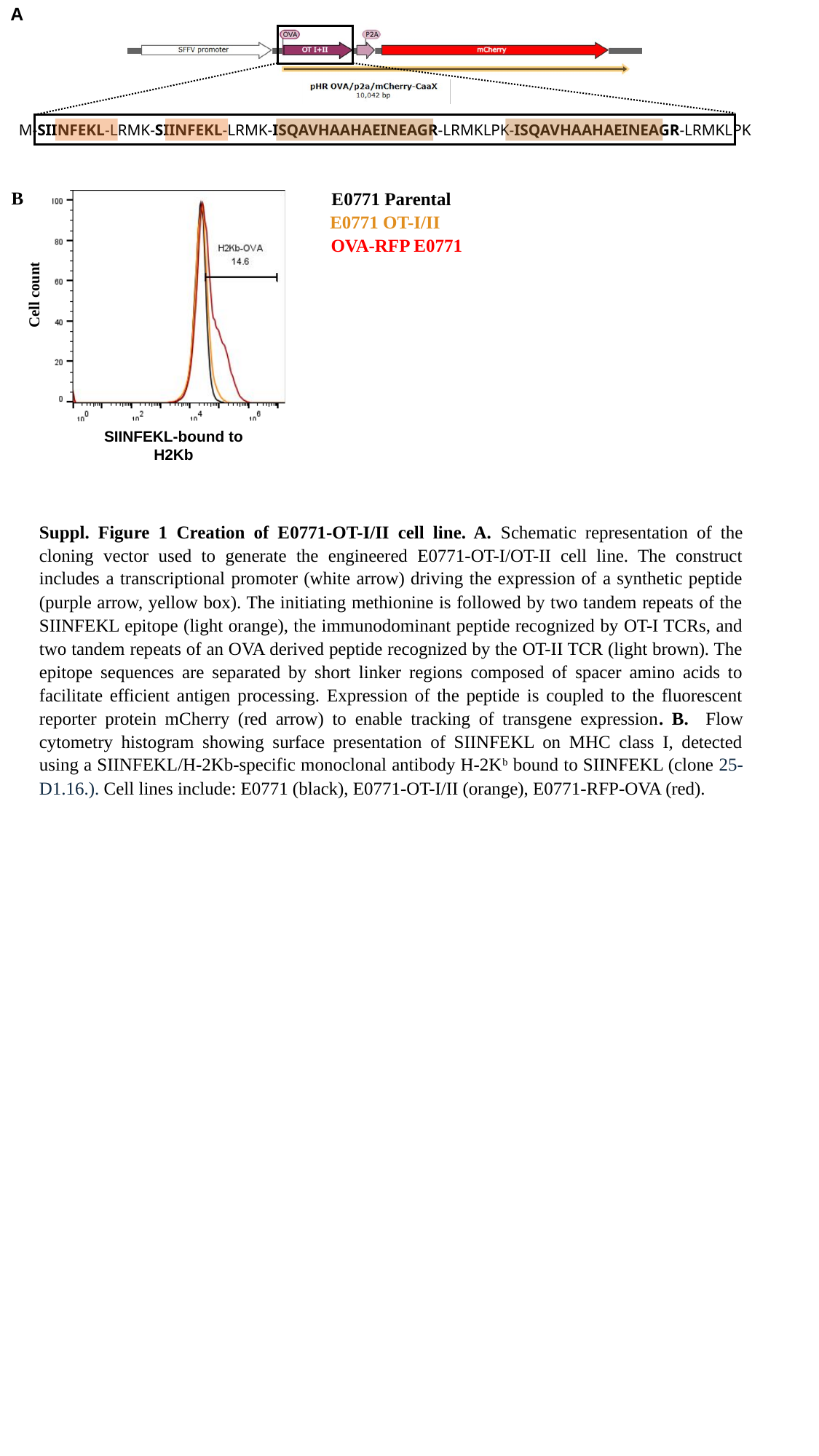

A
M-SIINFEKL-LRMK-SIINFEKL-LRMK-ISQAVHAAHAEINEAGR-LRMKLPK-ISQAVHAAHAEINEAGR-LRMKLPK
B
E0771 Parental
Cell count
SIINFEKL-bound to H2Kb
E0771 OT-I/II
OVA-RFP E0771
Suppl. Figure 1 Creation of E0771-OT-I/II cell line. A. Schematic representation of the cloning vector used to generate the engineered E0771-OT-I/OT-II cell line. The construct includes a transcriptional promoter (white arrow) driving the expression of a synthetic peptide (purple arrow, yellow box). The initiating methionine is followed by two tandem repeats of the SIINFEKL epitope (light orange), the immunodominant peptide recognized by OT-I TCRs, and two tandem repeats of an OVA derived peptide recognized by the OT-II TCR (light brown). The epitope sequences are separated by short linker regions composed of spacer amino acids to facilitate efficient antigen processing. Expression of the peptide is coupled to the fluorescent reporter protein mCherry (red arrow) to enable tracking of transgene expression. B. Flow cytometry histogram showing surface presentation of SIINFEKL on MHC class I, detected using a SIINFEKL/H-2Kb-specific monoclonal antibody H-2Kb bound to SIINFEKL (clone 25-D1.16.). Cell lines include: E0771 (black), E0771-OT-I/II (orange), E0771-RFP-OVA (red).

## Slide 2
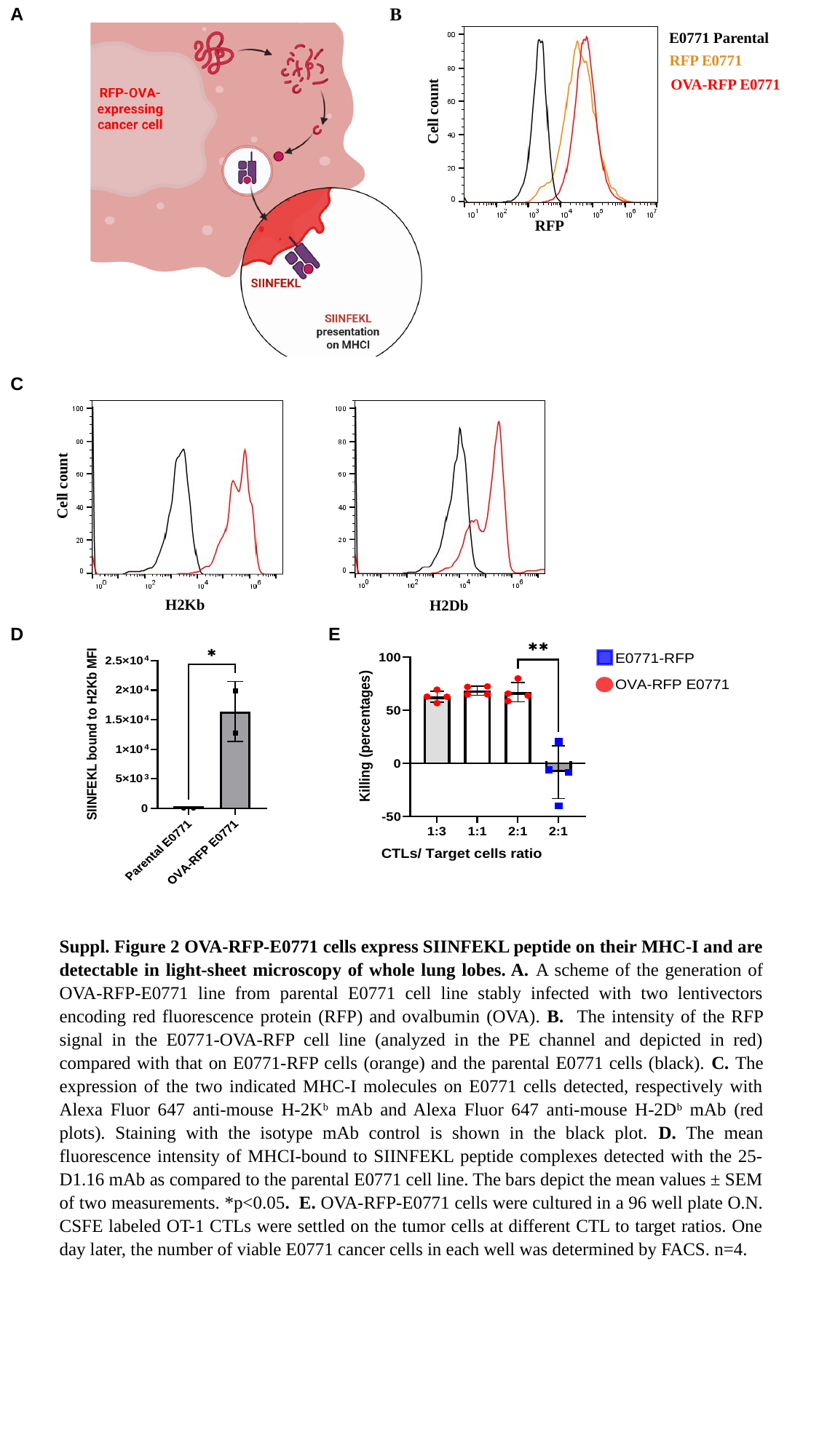

A
B
E0771 Parental
Cell count
RFP
RFP E0771
OVA-RFP E0771
C
H2Db
Cell count
H2Kb
D
E
Suppl. Figure 2 OVA-RFP-E0771 cells express SIINFEKL peptide on their MHC-I and are detectable in light-sheet microscopy of whole lung lobes. A. A scheme of the generation of OVA-RFP-E0771 line from parental E0771 cell line stably infected with two lentivectors encoding red fluorescence protein (RFP) and ovalbumin (OVA). B. The intensity of the RFP signal in the E0771-OVA-RFP cell line (analyzed in the PE channel and depicted in red) compared with that on E0771-RFP cells (orange) and the parental E0771 cells (black). C. The expression of the two indicated MHC-I molecules on E0771 cells detected, respectively with Alexa Fluor 647 anti-mouse H-2Kb mAb and Alexa Fluor 647 anti-mouse H-2Db mAb (red plots). Staining with the isotype mAb control is shown in the black plot. D. The mean fluorescence intensity of MHCI-bound to SIINFEKL peptide complexes detected with the 25-D1.16 mAb as compared to the parental E0771 cell line. The bars depict the mean values ± SEM of two measurements. *p<0.05. E. OVA-RFP-E0771 cells were cultured in a 96 well plate O.N. CSFE labeled OT-1 CTLs were settled on the tumor cells at different CTL to target ratios. One day later, the number of viable E0771 cancer cells in each well was determined by FACS. n=4.

## Slide 3
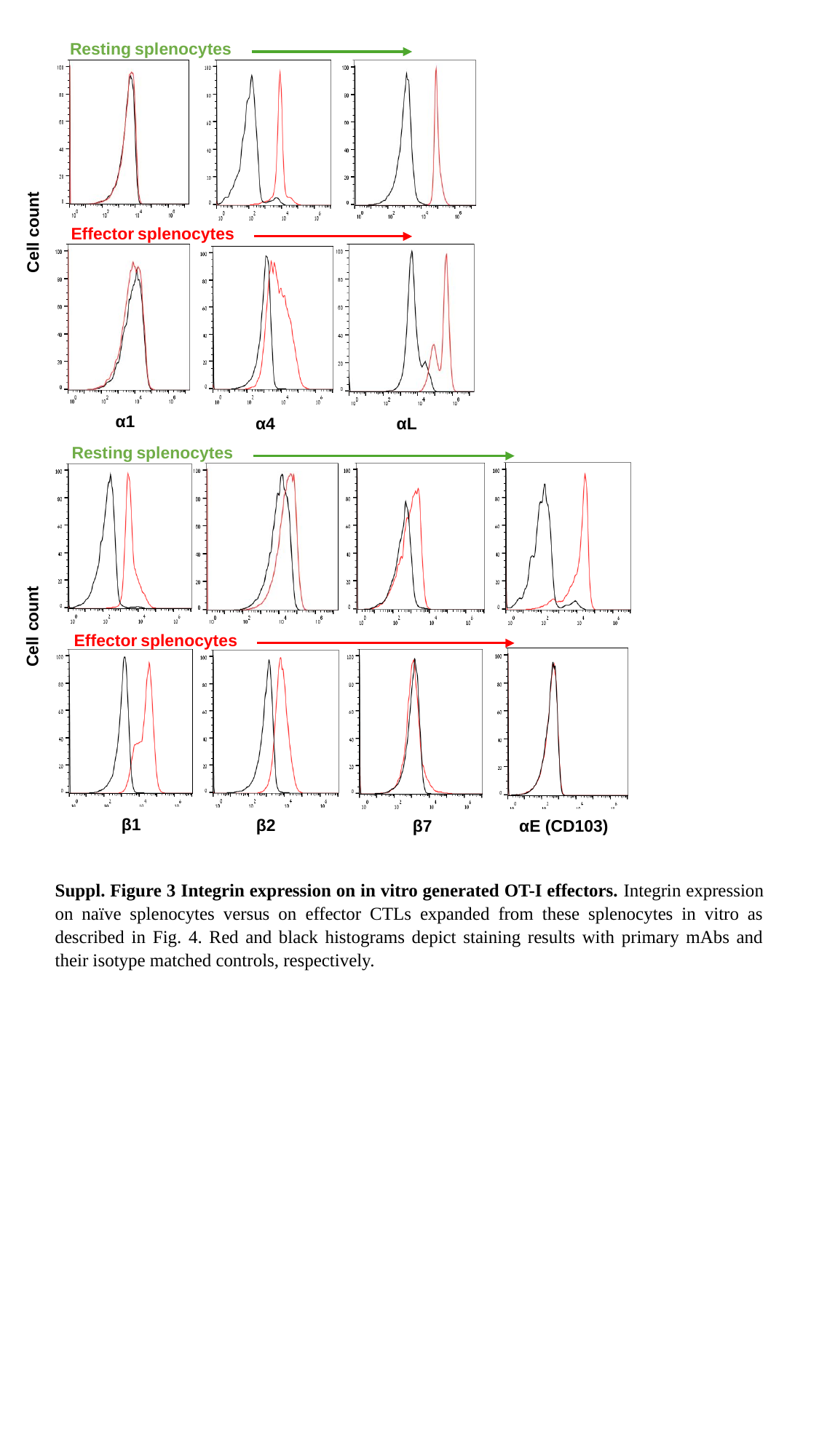

Resting splenocytes
Cell count
α1
αL
α4
Cell count
β1
β2
β7
αE (CD103)
Effector splenocytes
Resting splenocytes
Effector splenocytes
Suppl. Figure 3 Integrin expression on in vitro generated OT-I effectors. Integrin expression on naïve splenocytes versus on effector CTLs expanded from these splenocytes in vitro as described in Fig. 4. Red and black histograms depict staining results with primary mAbs and their isotype matched controls, respectively.

## Slide 4
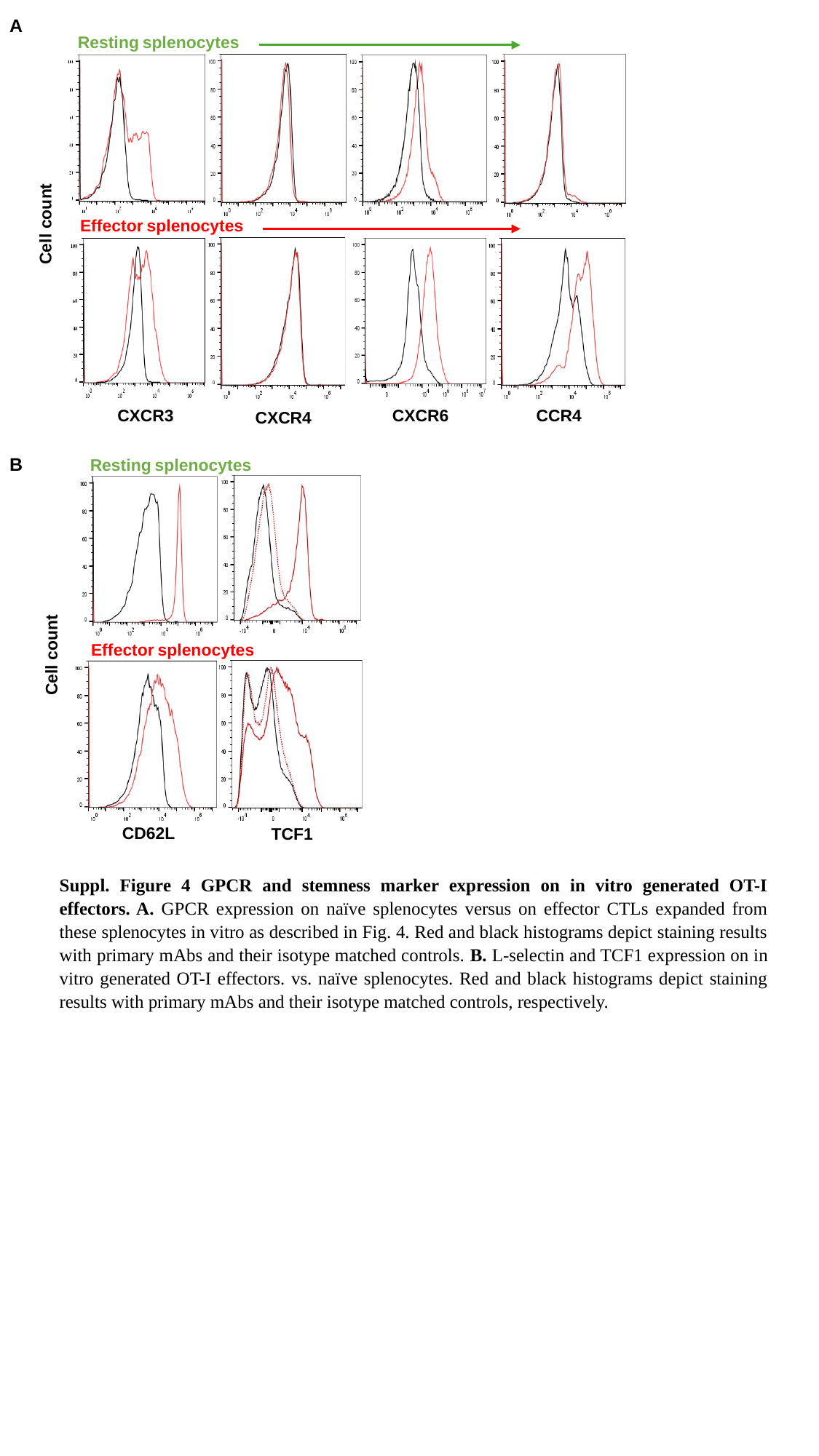

A
Resting splenocytes
Cell count
Effector splenocytes
CXCR3
CCR4
CXCR6
CXCR4
B
Resting splenocytes
Cell count
CD62L
TCF1
Effector splenocytes
Suppl. Figure 4 GPCR and stemness marker expression on in vitro generated OT-I effectors. A. GPCR expression on naïve splenocytes versus on effector CTLs expanded from these splenocytes in vitro as described in Fig. 4. Red and black histograms depict staining results with primary mAbs and their isotype matched controls. B. L-selectin and TCF1 expression on in vitro generated OT-I effectors. vs. naïve splenocytes. Red and black histograms depict staining results with primary mAbs and their isotype matched controls, respectively.

## Slide 5
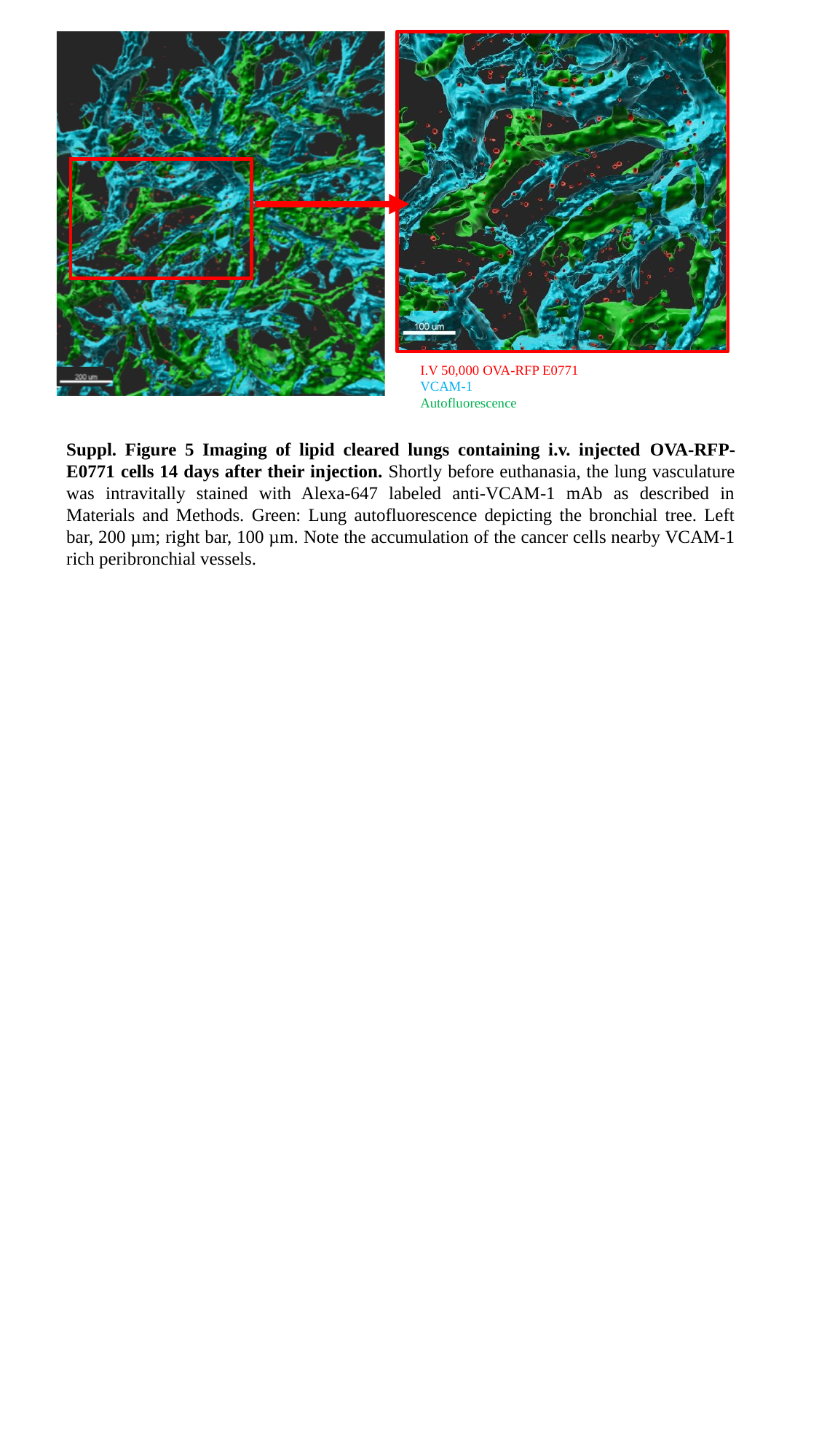

I.V 50,000 OVA-RFP E0771
VCAM-1
Autofluorescence
Suppl. Figure 5 Imaging of lipid cleared lungs containing i.v. injected OVA-RFP-E0771 cells 14 days after their injection. Shortly before euthanasia, the lung vasculature was intravitally stained with Alexa-647 labeled anti-VCAM-1 mAb as described in Materials and Methods. Green: Lung autofluorescence depicting the bronchial tree. Left bar, 200 µm; right bar, 100 µm. Note the accumulation of the cancer cells nearby VCAM-1 rich peribronchial vessels.

## Slide 6
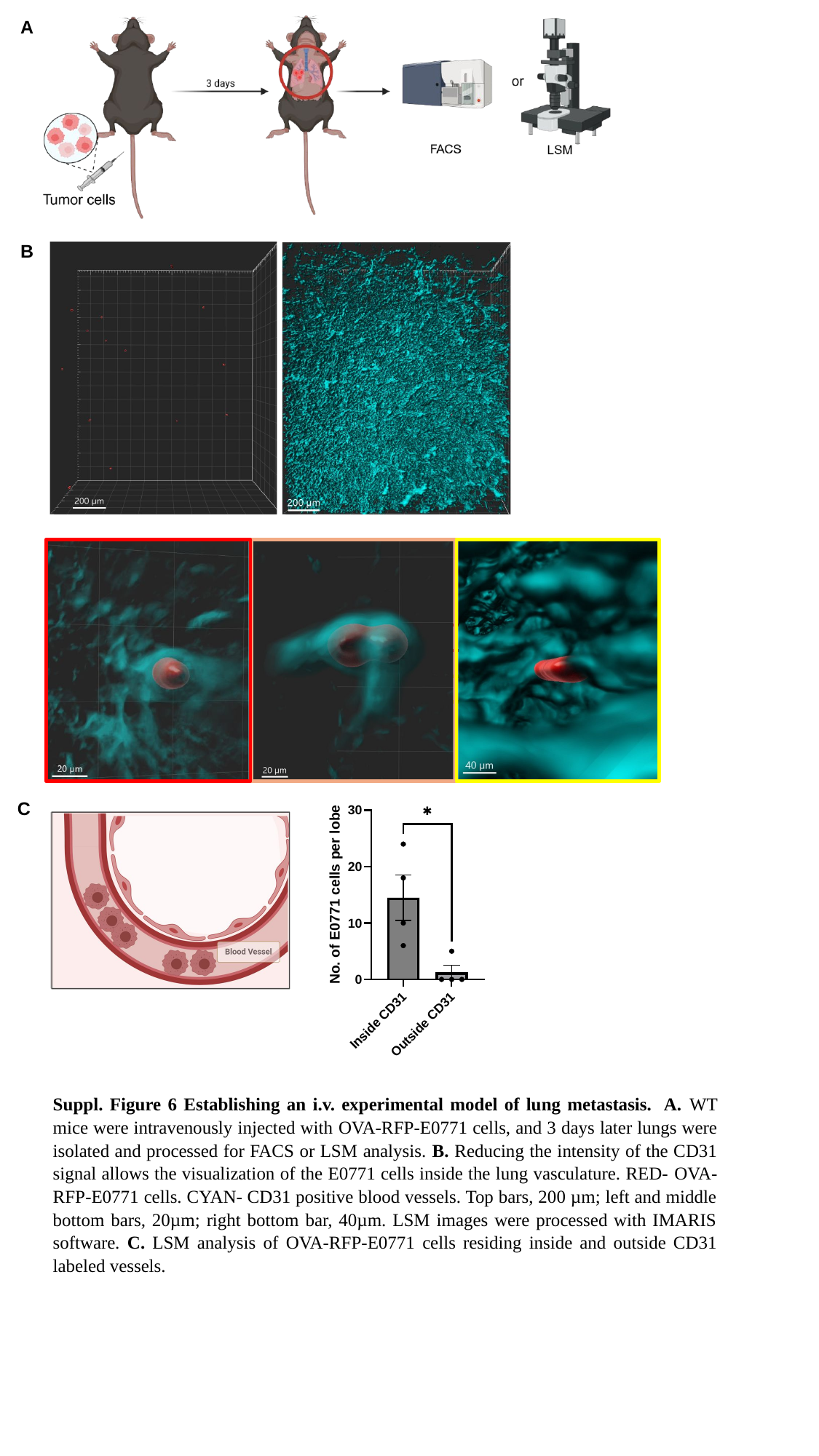

A
B
C
Suppl. Figure 6 Establishing an i.v. experimental model of lung metastasis. A. WT mice were intravenously injected with OVA-RFP-E0771 cells, and 3 days later lungs were isolated and processed for FACS or LSM analysis. B. Reducing the intensity of the CD31 signal allows the visualization of the E0771 cells inside the lung vasculature. RED- OVA-RFP-E0771 cells. CYAN- CD31 positive blood vessels. Top bars, 200 µm; left and middle bottom bars, 20µm; right bottom bar, 40µm. LSM images were processed with IMARIS software. C. LSM analysis of OVA-RFP-E0771 cells residing inside and outside CD31 labeled vessels.

## Slide 7
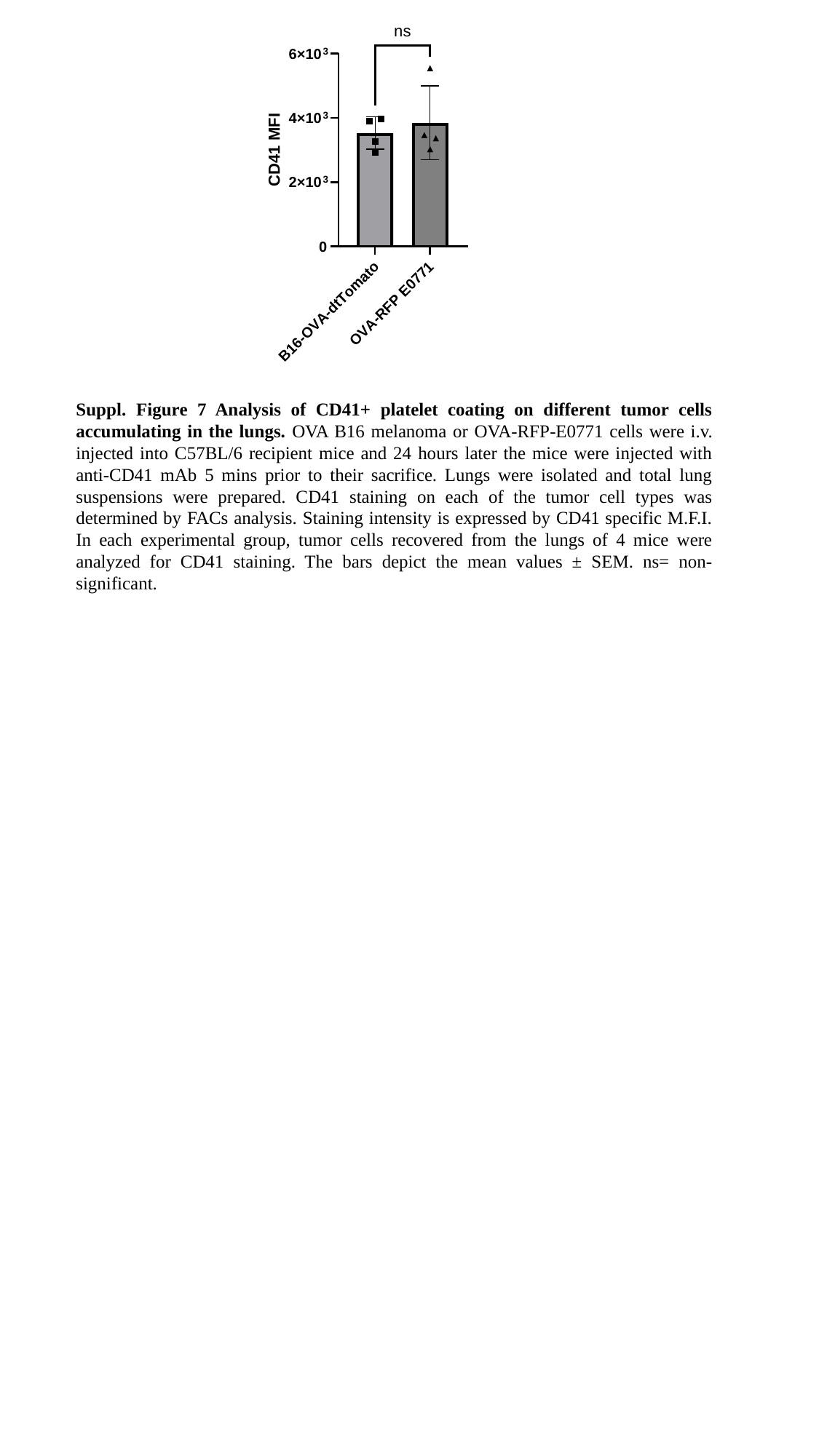

Suppl. Figure 7 Analysis of CD41+ platelet coating on different tumor cells accumulating in the lungs. OVA B16 melanoma or OVA-RFP-E0771 cells were i.v. injected into C57BL/6 recipient mice and 24 hours later the mice were injected with anti-CD41 mAb 5 mins prior to their sacrifice. Lungs were isolated and total lung suspensions were prepared. CD41 staining on each of the tumor cell types was determined by FACs analysis. Staining intensity is expressed by CD41 specific M.F.I. In each experimental group, tumor cells recovered from the lungs of 4 mice were analyzed for CD41 staining. The bars depict the mean values ± SEM. ns= non-significant.

## Slide 8
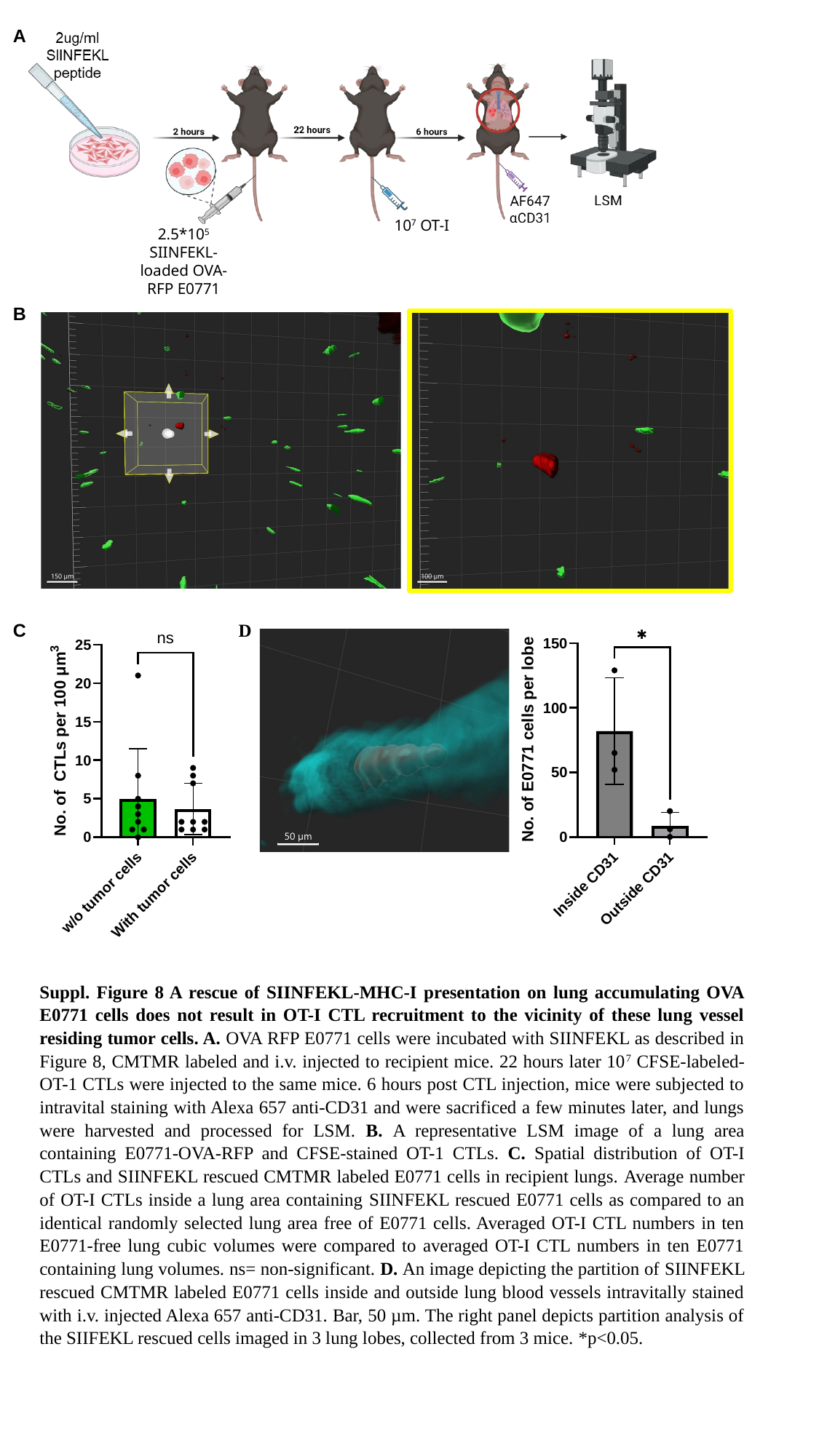

A
107 OT-I
2.5*105 SIINFEKL-loaded OVA-RFP E0771
B
C
D
Suppl. Figure 8 A rescue of SIINFEKL-MHC-I presentation on lung accumulating OVA E0771 cells does not result in OT-I CTL recruitment to the vicinity of these lung vessel residing tumor cells. A. OVA RFP E0771 cells were incubated with SIINFEKL as described in Figure 8, CMTMR labeled and i.v. injected to recipient mice. 22 hours later 107 CFSE-labeled-OT-1 CTLs were injected to the same mice. 6 hours post CTL injection, mice were subjected to intravital staining with Alexa 657 anti-CD31 and were sacrificed a few minutes later, and lungs were harvested and processed for LSM. B. A representative LSM image of a lung area containing E0771-OVA-RFP and CFSE-stained OT-1 CTLs. C. Spatial distribution of OT-I CTLs and SIINFEKL rescued CMTMR labeled E0771 cells in recipient lungs. Average number of OT-I CTLs inside a lung area containing SIINFEKL rescued E0771 cells as compared to an identical randomly selected lung area free of E0771 cells. Averaged OT-I CTL numbers in ten E0771-free lung cubic volumes were compared to averaged OT-I CTL numbers in ten E0771 containing lung volumes. ns= non-significant. D. An image depicting the partition of SIINFEKL rescued CMTMR labeled E0771 cells inside and outside lung blood vessels intravitally stained with i.v. injected Alexa 657 anti-CD31. Bar, 50 µm. The right panel depicts partition analysis of the SIIFEKL rescued cells imaged in 3 lung lobes, collected from 3 mice. *p<0.05.

## Slide 9
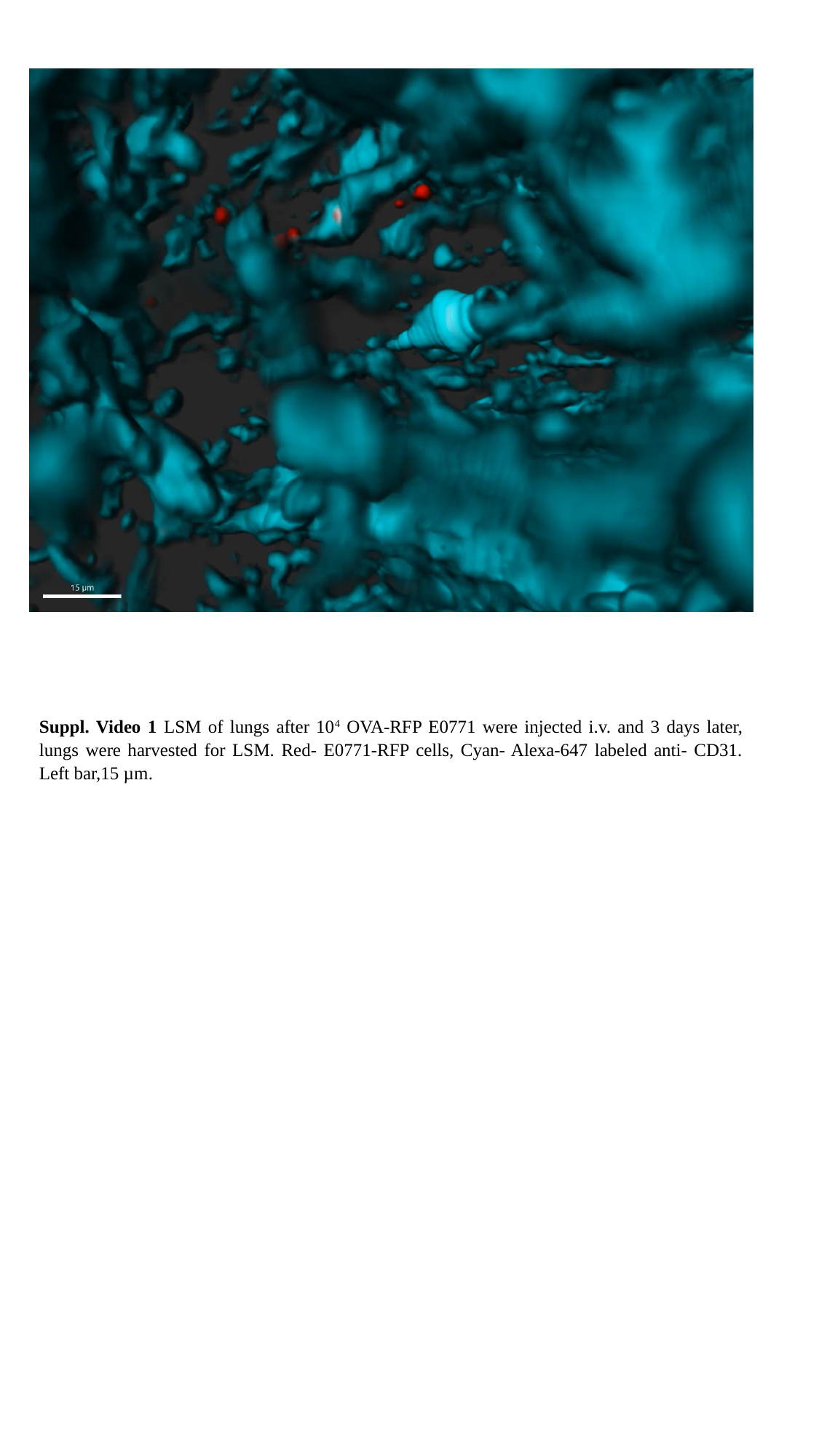

Suppl. Video 1 LSM of lungs after 104 OVA-RFP E0771 were injected i.v. and 3 days later, lungs were harvested for LSM. Red- E0771-RFP cells, Cyan- Alexa-647 labeled anti- CD31. Left bar,15 µm.

## Slide 10
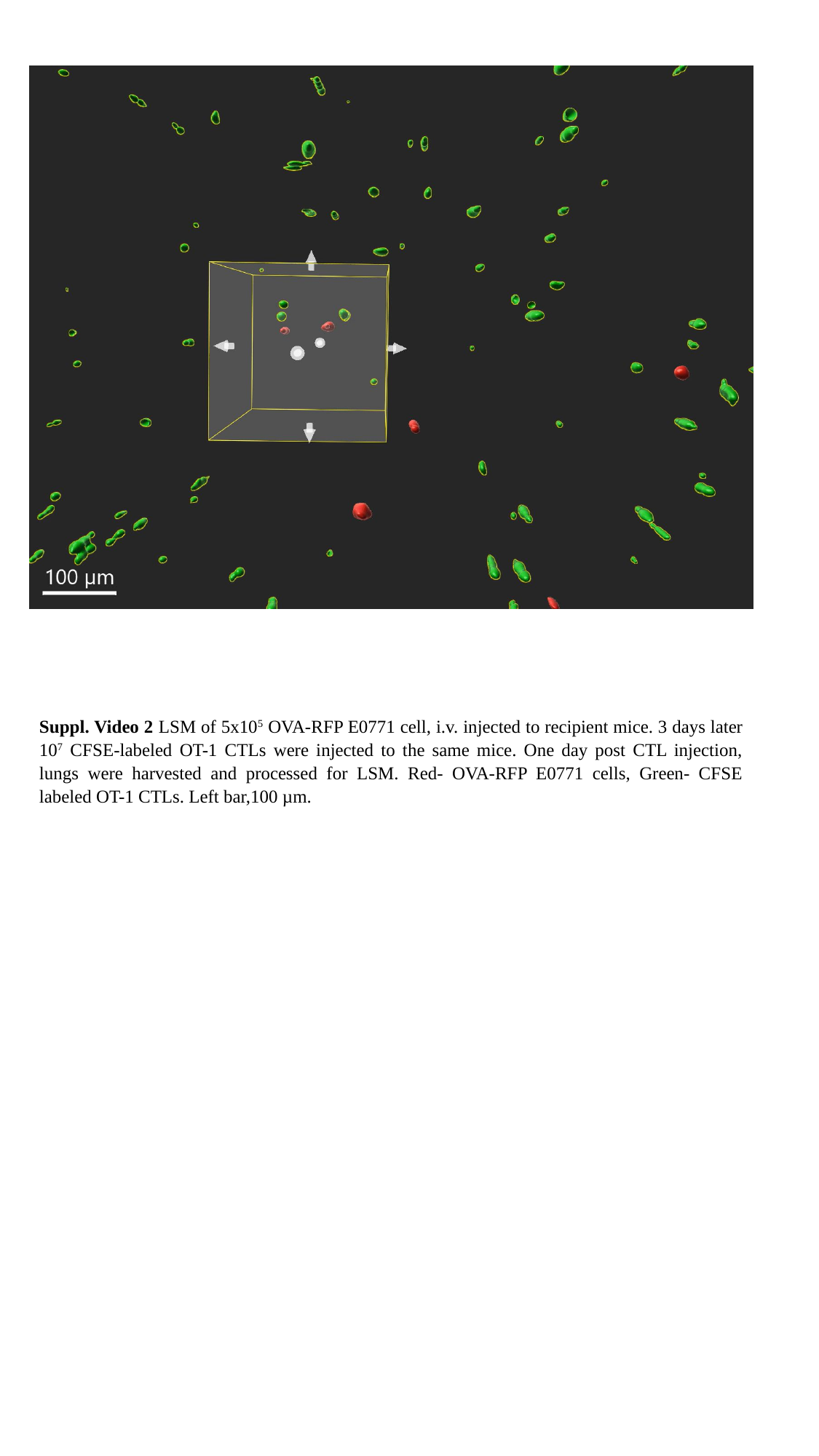

Suppl. Video 2 LSM of 5x10^5^ OVA-RFP E0771 cell, i.v. injected to recipient mice. 3 days later 107 CFSE-labeled OT-1 CTLs were injected to the same mice. One day post CTL injection, lungs were harvested and processed for LSM. Red- OVA-RFP E0771 cells, Green- CFSE labeled OT-1 CTLs. Left bar,100 µm.
